# Evaluation of Fused Pyrrolothiazole Systems as Correctors of Mutant CFTR Protein

**DOI:** 10.3390/molecules26051275

**Published:** 2021-02-26

**Authors:** Virginia Spanò, Marilia Barreca, Vincenzo Cilibrasi, Michele Genovese, Mario Renda, Alessandra Montalbano, Luis Juan Vicente Galietta, Paola Barraja

**Affiliations:** 1 Department of Biological, Chemical, and Pharmaceutical Sciences and Technologies (STEBICEF), University of Palermo, Via Archirafi 32, 90123 Palermo, Italy; virginia.spano@unipa.it (V.S.); marilia.barreca@unipa.it (M.B.); vincenzocilibrasi@gmail.com (V.C.); paola.barraja@unipa.it (P.B.); 2 Telethon Institute of Genetics and Medicine (TIGEM), Campi Flegrei 34, 80078 Naples, Italy; m.genovese@tigem.it (M.G.); m.renda@tigem.it (M.R.); l.galietta@tigem.it (L.J.V.G.); 3 Department of Translational Medical Sciences (DISMET), University of Naples, “Federico II”, Via Sergio Pansini 5, 80131 Naples, Italy

**Keywords:** cystic fibrosis, CFTR, F508del-CFTR, CFTR corrector, CFTR potentiator

## Abstract

Cystic fibrosis (CF) is a genetic disease caused by mutations that impair the function of the CFTR chloride channel. The most frequent mutation, F508del, causes misfolding and premature degradation of CFTR protein. This defect can be overcome with pharmacological agents named “correctors”. So far, at least three different classes of correctors have been identified based on the additive/synergistic effects that are obtained when compounds of different classes are combined together. The development of class 2 correctors has lagged behind that of compounds belonging to the other classes. It was shown that the efficacy of the prototypical class 2 corrector, the bithiazole corr-4a, could be improved by generating conformationally-locked bithiazoles. In the present study, we investigated the effect of tricyclic pyrrolothiazoles as analogues of constrained bithiazoles. Thirty-five compounds were tested using the functional assay based on the halide-sensitive yellow fluorescent protein (HS-YFP) that measured CFTR activity. One compound, having a six atom carbocyle central ring in the tricyclic pyrrolothiazole system and bearing a pivalamide group at the thiazole moiety and a 5-chloro-2-methoxyphenyl carboxamide at the pyrrole ring, significantly increased F508del-CFTR activity. This compound could lead to the synthesis of a novel class of CFTR correctors.

## 1. Introduction

**Figure 1:**
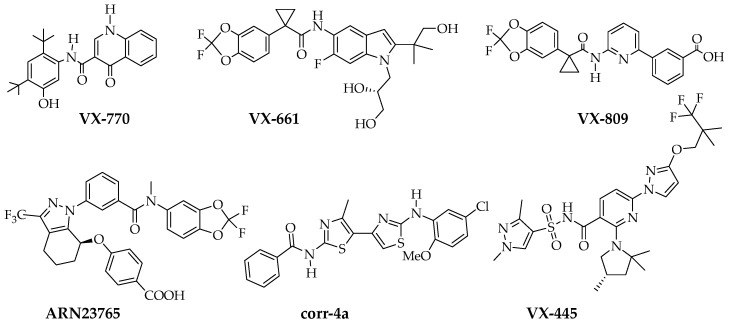
Structures of VX-770 (potentiator), VX-809 (Class I corrector), VX-661 (Class I corrector), ARN23765 (Class I corrector), corr-4a (Class II corrector) and VX-445 (Class III corrector).

Cystic fibrosis (CF), one of the most frequent genetic diseases [1], is caused by mutations that impair the expression and function of CFTR chloride channel. CF is a multi-organ pathology, with particularly severe consequences on the lungs, pancreas, and liver [1,2,3]. Pharmacological rescue of mutant CFTR function has become an effective therapeutic strategy to correct the basic defect in a large cohort of CF patients [4,5]. However, the therapy of CF with CFTR pharmacological modulators has to be tailored to the specific mutations carried by each patient. For example, Kalydeco, whose active principle is the “potentiator” VX-770 (Figure 1), is highly effective on patients carrying at least one copy of missense mutations causing CFTR channel gating defects [6,7,8]. Instead, additional types of small molecules, called “correctors”, need to be used to target F508del, the most frequent mutation among CF patients [9]. This mutation causes multiple folding and stability defects in CFTR protein resulting in premature degradation by the ubiquitin-proteasome cell system [10,11]. Highly effective rescue of F508del-CFTR mutant requires combinations of correctors. Correctors have been grouped in three classes according to the additive/synergistic effects that are generated when compounds belonging to separate classes are combined together [11,12]. VX-809 (Figure 1) [13], also known as lumacaftor, the first corrector to be approved for therapeutic use in CF patients, belongs to class 1. It has been postulated that these compounds act by targeting one of the problems caused by F508del, i.e., defective domain-domain interaction [10,11,12]. Other types of class 1 correctors are VX-661 (Figure 1), known as tezacaftor, which is already included in drugs approved for CF patients [14], and ARN23765 (Figure 1) [15]. Class 3 correctors are believed to target NBD1, the domain where F508del is localized [11,12]. VX-445 (elexacaftor) (Figure 1), a highly effective corrector that is now included in triple drug combinations together with VX-770 and VX-661 [16], has been recently classified as a class 3 compound [17]. Class 2 correctors, such as the bithiazole corr-4a (Figure 1) [18], are believed to act with a different mechanism with respect to class 1 and class 3 compounds [11,12]. Although corr-4a was one of the first correctors to be discovered, the development of class 2 correctors into drugs has lagged behind that of other types of correctors. Development of additional type 2 correctors, with the ability to synergize with class 1 and class 3 compounds, would be highly desirable to design novel combinatorial treatments for CF patients.

**Figure 2:**
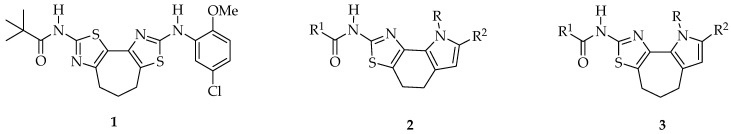
Structure of *N*-(8-((5-chloro-2-methoxyphenyl)amino)-5,6-dihydro-4*H*cyclohepta[1,2-*d*:3,4-*d′*] bis(thiazole)-2-yl)pivalamidebithiazole (**1**), [1,3]thiazolo[5,4-*g*]indole-2-amines (**2**) and pyrrolo[3′,2′:6,7]cyclohepta[1,2-*d*][1,3]thiazol-2-amines (**3**).

To generate corr-4a derivatives with improved corrector activity, conformationally-locked analogues were previously investigated [19,20]. Thus, by locking the thiazole core into a *s*-cis conformation, constrained bithiazoles of type **1** (Figure 2) bearing a central ring ranging from 5 to 8 carbon units were obtained [19,20]. In vitro data, on F508del-CFTR cells, suggested that modulating the constraining ring size in these correctors did not significantly enhance their potency (IC_50_), but significantly affected maximum efficacy. The best compound **1**, bearing a cyclohepta ring between the two thiazole moieties, showed a 1.5-fold improved correction compared to free rotable benchmark bithiazole [20]. In particular, structural modifications revealed a correlation between the corrector activity and the presence of the amide and aniline groups into the bithiazole scaffold. The best combination in terms of potency and efficacy was achieved when 5-chloro-2-methoxyphenyl and the pivalamide groups were incorporated: The resulting compound had improved half-effective concentration (2.3 vs. 6 μM) and maximal effect (678 vs. 475 μM/s) with respect to corr-4a. The increase in Vmax is indicative of a higher number of F508del-CFTR proteins trafficking to the plasma membrane. It was postulated that the cyclohepta ring provides the required flexibility in the bithiazole core during protein binding to the active site.

Pyrroles are valuable heterocycles in medicinal chemistry, and offer a high degree of structural diversity as a useful tool for developing new therapeutic agents.

For many years, we have been involved in studies dealing with nitrogen heterocycles and much of our attention has been paid to tricyclic pyrrolo-fused systems [21,22,23,24,25,26,27,28,29,30,31,32]. Based on our past experience, we decided to investigate the effect on corrector activity of the replacement of one thiazole unit with a pyrrole moiety, which could have allowed the incorporation of some common structural features with constrained bithiazoles, aiming at the identification of new correctors. Among all possible pyrrolothiazole derivatives, depending on condensation of the pyrrole unit into the tricyclic scaffold, we chose to maintain the nitrogen position of the parent structure. Thus, we prepared a focused set of pyrrolothiazoles, as analogues of constrained bithiazoles (Figure 2). We decided to start our exploration from new classes of pyrrolothiazoles of type **2** and **3** (Figure 2), incorporating a carbocycle central ring ranging from six to seven carbon atoms and the most relevant structural features such as the 5-chloro-2-methoxyphenyl and the pivalamide groups, that would retain many aspects of the core framework of compounds **1**.

## 2. Results and Discussion

### 2.1. Chemistry

**Scheme 1:**
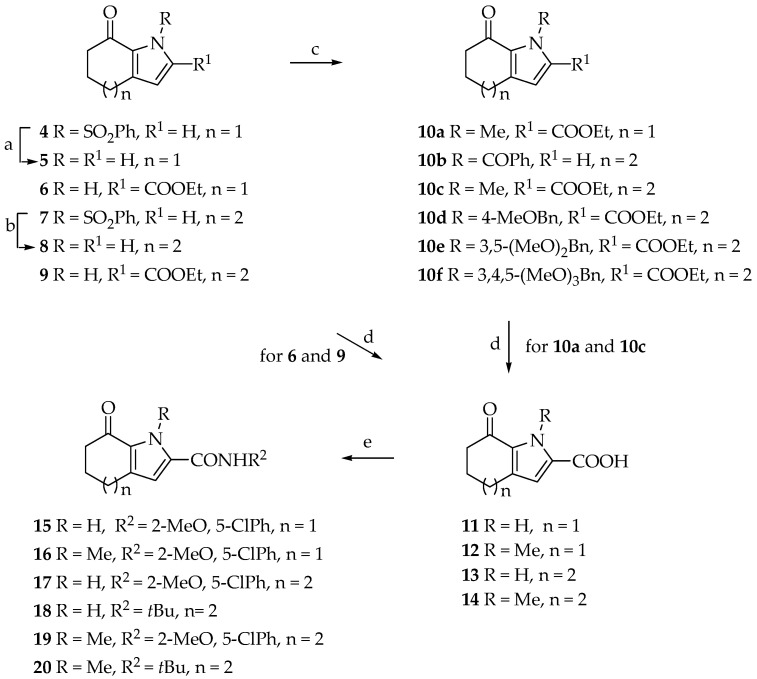
Synthesis of tetrahydroindol-7-ones **4**–**6**,**10a**,**11**,**12**,**15**,**16** and tetrahydrocycloheptapyrrol-8-ones **7**–**9**,**10b**–**f**,**13**,**14**,**17**–**20**. Reagents and conditions. (**a**) KOH, ethanol, reflux, 2 h, 88%; (**b**) NaOH, ethanol, reflux, 3 h, 80%; (**c**) NaH, DMF, 0 °C to rt, 1.5 h then alkyl or aralkylhalide, 0 °C to rt, 2–24 h, 80–96%; (**d**) KOH, ethanol, reflux, 0.5–1.5 h, 71–86%; (**e**) HOBt, DIPEA, EDC, DMF, rt, 10 min then 1-(5-chloro-2-methoxyphenyl)amine or *tert*-butylamine, rt or 100 °C, 3–20 h, 60–72%.

**Scheme 2:**
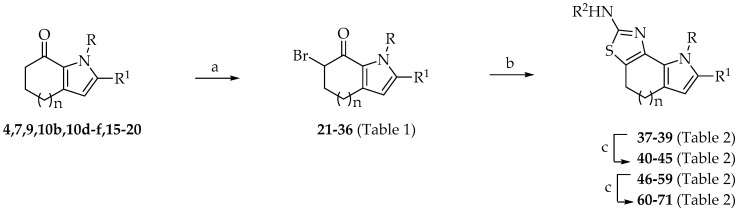
**[1,3]**Thiazolo[5,4-*g*]indole-2-amines **37**–**45** and pyrrolo[3′,2′:6,7]cyclohepta[1,2-*d*][1,3]thiazole-2-amines **46**–**71**. Reagents and conditions. (**a**) Method A: CuBr_2_, ethyl acetate, reflux, 2 h, or Method B: pyridine hydrobromide perbromide, THF, rt, 16 h, 60–97%; (**b**) substituted thiourea, Na_2_CO_3_, DMF, rt, 16 h, 53–96%; (**c**) acetyl chloride, DIPEA, DMF, rt, 24 h, (for derivatives **60**–**65**), 59–73% or acyl chloride, Et_3_N, 1,4-dioxane, rt, 16 h, (for derivatives **40**–**45**, **66**–**71**), 60–81%.

The synthetic strategy used for the preparation of the starting ketones (Scheme 1, Table 1) and of the desired tricyclic ring systems is depicted in Scheme 2 and final compounds reported in Table 2. α-Bromo-ketones (Table 1) were used as key intermediates suitable for the anellation of the thiazole ring upon reaction with thioureas, under typical Hantzsch reaction conditions.

#### Synthesis of [1,3]Thiazolo[5,4-*g*]Indole-2-Amines (**37**–**45**) and Pyrrolo[3′,2′:6,7]Cyclohepta[1,2-*d*][1,3]Thiazole-2-Amines (**46**–**71**)

We started from the synthesis of tetrahydroindol-7-ones **4**–**6** (*n* = 1) and tetrahydrocycloheptapyrrol-8-ones **7**–**9** (*n* = 2) (Scheme 1), to achieve the target [1,3]thiazoles [33,34,35].

Ketones **5**,**6**,**8**,**9** were functionalized at the pyrrole nitrogen using the suitable alkyl or aryl halide in the presence of sodium hydride to give the corresponding derivatives **10a–f** (80–96%) (Scheme 1). In order to mimic the decoration of most active constrained bithiazole derivatives, the ethoxycarbonyl group of ketones **6**,**9** and **10a**,**10c** was converted into carboxyamide. Thus, hydrolisis with potassium hydroxyde in refluxing ethanol gave carboxy acid derivatives **11**–**14** in excellent yield (71–86%) (Scheme 1), which were further reacted with the suitable amine in the presence of 1-hydroxy-benzotriazole (HOBt) and *N*-Ethyl-*N*′-(3-dimethylaminopropyl)carbodiimide hydrochloride (EDC), as activating agent, allowing the isolation of the corresponding carboxamide derivatives **15**–**20** (60–72%) (Scheme 1).

α-Bromination of ketones **4**,**7**,**9**,**10b**,**10d**–**f**,**15**–**20** into α-bromo-derivatives **21**–**36** was achieved by two different methods (Table 1). Copper(II)bromide was used to obtain α-bromo-ketones **21**,**23**,**25**–**30** (Method A) or pyridine hydrobromide perbromide (Method B) to achieve **24**,**31**–**36** (60–97%) (Scheme 2, Table 1).

In the case of ketones bearing an ethoxycarbonyl or a carboxyamide group at the pyrrole ring, the corresponding α-bromo-ketones were achieved in high yields (65–97%) (Scheme 2, Table 1).

However, bromination of **10e**, beside the desired α-bromo-ketone **28**, led also to **30** as a by-product, bearing a bromine at the aryl moiety (Scheme 2, Table 1). The α-bromo-ketone **22** was obtained in good yield (72%) by deprotection of N-benzoyl derivatives **24** (Scheme 2, Table 1).

α-Bromo-ketones **21**–**36** were cyclized with thiourea or the suitable substituted thioureas, in presence of sodium carbonate, allowing the isolation of [1,3]thiazoles **37**–**39** and **46**–**59** from moderate to excellent yield (53–96%) (Scheme 2, Table 2). Finally, [1,3]thiazoles, bearing an amino group in position 2 of the tricyclic system, were subjected to acylation with the proper acyl chloride (acetyl chloride, trimethylacetyl chloride, isobutyryl chloride) in presence of N,N-diisopropylethylamine (DIPEA) or triethylamine to give compounds **40**–**45** (60–81%) and **60**–**71** (59–80%) (Scheme 2, Table 2).

### 2.2. Biology

**Figure 3:**
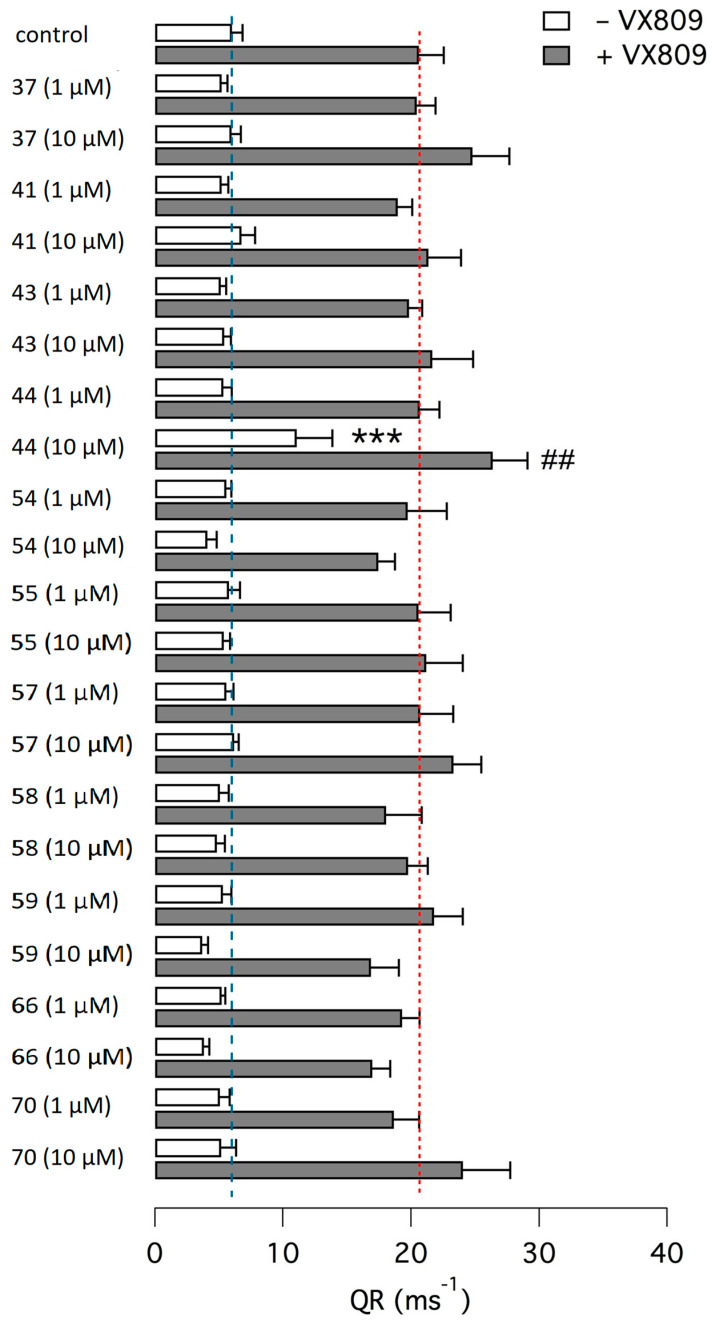
Functional evaluation of CFTR correctors. The bar graph reports the quenching rate (QR), i.e., the anion transport mediated by F508del-CFTR in the plasma membrane, as determined with the HS-YFP functional assay. Before the assay, CFBE41o-cells were treated for 24 h with indicated compounds at 1 and 10 µM, with and without VX-809 (1 µM). Results were compared to those of cells treated with vehicle (DMSO) or VX-809 (1 µM) alone. Each bar reports the average and SD from six experiments. ***, *p* < 0.001 vs. control (no VX-809). ##, *p* < 0.01 vs. VX-809 alone.

All compounds were tested in a CFTR corrector assay that is carried out on CFBE41o-cells expressing F508del-CFTR. Such cells also express the halide-sensitive yellow fluorescent protein (HS-YFP). CFTR-dependent iodide influx causes (HS-YFP) fluorescence quenching. Hence, the quenching rate is proportional to the amount of CFTR channels in the plasma membrane and therefore reflects the efficacy of correctors in rescuing mutant CFTR from intracellular compartments. Cells were incubated for 24 h with compounds at 1 and 10 µM. Treatment was also done in combination with VX-809 (1 µM). After treatment, cells were further stimulated with forskolin and genistein to fully activate CFTR in the plasma membrane and promote anion transport. A representative set of data is shown in Figure 3. As expected, incubation with the control corrector VX-809 elicited a nearly three-fold increase in anion transport that is the result of improved trafficking of mutant CFTR from intracellular compartments to plasma membrane. Among all compounds tested, only compound **44** showed at 10 µM a significant effect, corresponding to 36% of VX-809 activity. Interestingly, combination of **44** with VX-809 elicited a significant additive effect (Figure 3).

From a structure activity point of view a six atom carbocyle ring, a pivalamide group and a 5-chloro-2-methoxyphenyl carboxamide at the thiazole and pyrrole rings respectively are required for corrector activity. In fact, moving to cyclohepta analogues a decrease of activity was observed (compare **44** with **70**). Any manipulation of peripheral groups in the pyrrolothiazole structure investigated led either to a decrease of the activity, as in the case of the isopropyl amide analogue **41**, or to a loss of activity.

**Figure 4:**
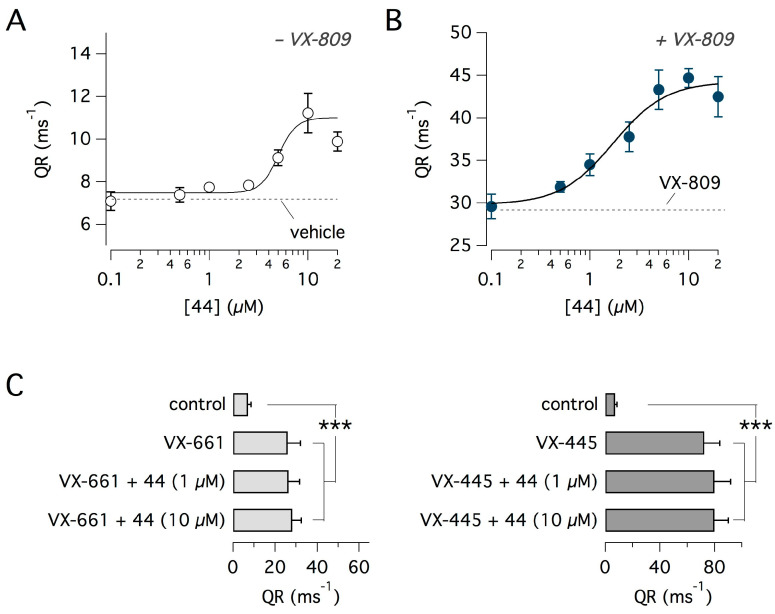
Characterization of compound **44**. (**A**,**B**) Dose-response relationships obtained by incubating cells with different concentrations of compound **44** in the absence (**A**) and presence (**B**) of VX-809 at 1 µM. After incubation, F508del-CFTR activity in the plasma membrane (i.e., anion transport reported as QR) was determined with the HS-YFP assay. Each symbol shows mean ± SD from six separate experiments. Fitting of data with a Hill equation showed a half-effective concentration of 5 and 1.7 µM in the absence and presence of VX-809, respectively. (**C**) Effect of combinations of compound **44** (at 1 and 10 µM) with 5 µM VX-661 (left) or 5 µM VX-445 (right). The two types of combinations did not result in additive effects. Each bar is the mean ± SD of eight experiments. ***, *p* <0.001 vs. control.

To assess potency and efficacy of compound **44**, we treated cells for 24 h at different concentrations in the range 0.1–20 µM. Treatment was done in the absence and in the presence of VX-809 at 1 µM. The resulting dose-response relationships are shown in Figure 4A,B. Interestingly, the apparent affinity of compound **44** appeared to be increased in the presence of VX-809 since the half-effective concentration decreased from 5 to 1.7 µM (Figure 4A,B). We also tested compound 44 in combination with VX-661 and VX-809 at their maximally-effective concentrations (Figure 4C). Compound **44** showed no evidence of additivity with VX-661 and VX-445.

## 3. Materials and Methods

### 3.1. Chemistry

All melting points were taken on a Büchi melting point M-560 apparatus. IR spectra were determined in bromoform with a Shimadzu FT/IR 8400S spectrophotometer. ^1^H and ^13^C NMR spectra were measured at 200 and 50.0 MHz, respectively, in DMSO-*d_6_* or CDCl_3_ solution using a Bruker Avance II series 200 MHz spectrometer. Column chromatography was performed with Merck silica gel (230−400 mesh ASTM) or a Büchi Sepacor chromatography module (prepacked cartridge system). Elemental analyses (C, H, N) were within ±0.4% of theoretical values and were performed with a VARIO EL III elemental analyzer.

Compounds **4**–**9**, **10a**,**10c**–**f** were prepared according to our previous published procedures [33,34,35].

#### 3.1.1. Synthesis of [1,3]Thiazolo[5,4-*g*]Indole-2-Amines and Pyrrolo[3′,2′:6,7]Cyclohepta[1,2-*d*][1,3]Thiazole-2-Amines

##### General Procedure for the Synthesis of 1-(Benzoyl)-4,5,6,7-Tetrahydrocyclohepta[*b*]Pyrrol-8(1*H*)-one (**10b**)

To a solution of the suitable ketones **8** (6 mmol) in anhydrous DMF (12 mL), NaH (6.6 mmol) was added at 0 °C and the reaction was stirred for 1 h and 30 min at room temperature. Then, benzoyl chloride (9 mmol) was added at 0 °C, and the reaction mixture was stirred at room temperature for 3 h. Then the reaction mixture was poured onto ice and brine (40 mL), and the aqueous solution was extracted with dichloromethane (3 × 40 mL). The organic phase was dried over Na_2_SO_4_ and the solvent evaporated under reduced pressure. The crude product was purified by column chromatography (dichloromethane). Colorless oil; yield: 82%; IR: ν_max_ = 1699 (CO) 1645 (CO) cm^−1^; ^1^H NMR (200 MHz, CDCl_3_) δ 1.83–2.01 (m, 4H, 2 × CH_2_), 2.59 (t, *J* = 6.1 Hz, 2H, CH_2_), 2.81–2.90 (m, 2H, CH_2_), 6.13 (d, *J* = 2.4 Hz, 1H, H-3), 7.17 (d, *J* = 2.4 Hz, 1H, H-2), 7.40–7.73 (5H, m, Ar); ^13^C NMR (500 MHz, CDCl_3_) δ 22.4 (t), 26.0 (t), 27.6 (t), 42.1 (t), 112.4 (d), 128.2 (d), 128.5 (2 × d), 129.6 (2 × d), 130.1 (s), 133.2 (d), 133.8 (s), 137.2 (s), 169.0 (s), 192.1 (s). Anal. Calcd. for C_16_H_15_NO_2_: C, 75.87; H, 5.97; N, 5.53. Found: 76.03; H, 6.12; N, 5.44.

##### General Procedure for the Synthesis of 7-Oxo-4,5,6,7-Tetrahydro-1*H*-Indole-2-Carboxylic Acid (**11**,**12**) and 8-Oxo-1,4,5,6,7,8-Hexahydrocyclohepta[*b*]Pyrrole-2-Carboxylic Acid (**13**,**14**)

To a solution of suitable ketones **6**,**9**,**10a**,**10c** (6.4 mmol) in ethanol (25 mL) a solution of aq. 50% KOH (4.8 mL) was added and the reaction mixture was heated at reflux until the reaction was complete (TLC). After cooling, the solvent was removed under reduced pressure. The residue was added of water and the solution was acidified with HCl 6 M. The solid formed was collected by filtration and dried.

##### 7-Oxo-4,5,6,7-Tetrahydro-1*H*-Indole-2-Carboxylic Acid (**11**)

This compound was obtained by reaction of **6** after 35 min. White solid; yield 71%; mp: 280–281 °C; IR: ν_max_ = 3405 (NH), 3176 (OH), 1709 (CO), 1635 (CO) cm^−1^; ^1^H NMR (200 MHz, DMSO-d_6_) δ 1.94–2.06 (m, 2H, CH_2_), 2.43 (t, *J* = 5.9 Hz, 2H, CH_2_), 2.68 (t, *J* = 5.9 Hz, 2H, CH_2_), 6.61 (s, 1H, H-3), 12.25 (s, 1H, OH); ^13^C NMR (50 MHz, DMSO-d_6_) δ 22.5 (t), 24.5 (t), 38.2 (t), 112.8 (d), 128.4 (s), 130.3 (s), 135.1 (s), 161.7 (s), 188.7 (s). Anal calcd for C_9_H_9_NO_3_: C, 60.33; H, 5.06; N, 7.82. Found: C, 60.47; H, 4.88; N, 7.99.

##### 1-Methyl-7-Oxo-4,5,6,7-Tetrahydro-1*H*-Indole-2-Carboxylic Acid (**12**)

This compound was obtained by reaction of **10a** after 1 h and 30 min. White solid; yield 86%; mp: 280–281 °C; IR: ν_max_ = 3113 (OH), 1709 (CO), 1686 (CO) cm^−1^; ^1^H NMR (200 MHz, DMSO-d_6_) δ 1.90–2.03 (m, 2H, CH_2_), 2.46 (t, *J* = 6.0 Hz, 2H, CH_2_), 2.68 (t, *J* = 6.0 Hz, 2H, CH_2_), 4.13 (s, 3H, CH_3_), 6.67 (s, 1H, H-3), 13.01 (s, 1H, OH); ^13^C NMR (50 MHz, DMSO-d_6_) δ 22.9 (t), 24.1 (t), 34.1 (q), 39.5 (t), 113.9 (d), 128.4 (s), 129.8 (s), 134.6 (s), 162.0 (s), 190.1 (s). Anal calcd for C_10_H_11_NO_3_: C, 62.17; H, 5.74; N, 7.25. Found: C, 62.33; H, 5.59; N, 7.11.

##### 8-Oxo-1,4,5,6,7,8-Hexahydrocyclohepta[*b*]Pyrrole-2-Carboxylic Acid (**13**)

This compound was obtained by reaction of **9** after 40 min. White solid; yield 82%; mp: 280–281 °C; IR: ν_max_ = 3296 (NH), 3216 (OH), 1715 (CO), 1686 (CO) cm^−1^; ^1^H NMR (200 MHz, DMSO-d_6_) δ 1.72–1.86 (m, 4H, 2 × CH_2_), 2.62 (t, *J* = 5.7 Hz, 2H, CH_2_), 2.79 (t, *J* = 5.7 Hz, 2H, CH_2_), 6.63 (s, 1H, H-3), 11.59 (s, 1H, NH), 12.60 (s, 1H, OH); ^13^C NMR (50 MHz, DMSO-d_6_) δ 21.5 (t), 25.4 (t), 26.1 (t), 41.3 (t), 115.6 (d), 127.0 (s), 131.9 (s), 132.1 (s), 161.4 (s), 192.0 (s). Anal calcd for C_10_H_11_NO_3_: C, 62.17; H, 5.74; N, 7.25. Found: C, 61.97; H, 5.88; N, 7.08.

##### 1-Methyl-8-Oxo-1,4,5,6,7,8-Hexahydrocyclohepta[*b*]Pyrrole-2-Carboxylic Acid (**14**)

This compound was obtained by reaction of **12** after 45 min. White solid; yield 75%; mp: 280–281 °C; IR: ν_max_ = 3410 (OH), 1721 (CO), 1680 (CO) cm^−1^; ^1^H NMR (200 MHz, DMSO-d_6_) δ 1.63–1.79 (m, 4H, 2 × CH_2_), 2.63 (t, *J* = 5.1 Hz, 2H, CH_2_), 2.75 (t, *J* = 5.1 Hz, 2H, CH_2_), 4.06 (s, 3H, CH_3_), 6.70 (s, 1H, H-3), 12.91 (s, 1H, OH); ^13^C NMR (50 MHz, DMSO-d_6_) δ 20.8 (t), 24.5 (t), 24.9 (t), 34.3 (q), 41.4 (t), 116.5 (d), 127.3 (s), 132.8 (s), 133.2 (s), 162.0 (s), 194.1 (s). Anal calcd for C_11_H_13_NO_3_: C, 63.76; H, 6.32; N, 6.76. Found: C, 63.88; H, 6.19; N, 6.59.

##### General Procedure for the Synthesis of *N*-(Substituted)-7-Oxo-4,5,6,7-Tetrahydro-1*H*-Indole-2-Carboxamide (**15**,**16**) and *N*-(Substituted)-8-Oxo-1,4,5,6,7,8-Hexahydrocyclohepta[*b*]Pyrrole-2-Carboxamide (**17**–**20**)

To a suspension of the suitable acid derivatives **11**–**14** (1.9 mmol) in anhydrous DMF (6 mL) *N,N*-diisopropylethylamine (1.9 mL, 10.9 mmol), 1-hydroxybenzotriazole hydrate (0.41 g, 3.0 mmol), and EDC (0.53 g, 2.8 mmol) were added. The reaction mixture was stirred at room temperature for 10 min. Then the proper amine (7.6 mmol) was added in one portion, and the resulting suspension was stirred at room temperature or at 100 °C until the reaction was complete (TLC). Then the reaction mixture was poured onto crushed ice and the solid formed was collected by filtration, dried and used in the next step without further purification.

##### *N*-(5-Chloro-2-Methoxyphenyl)-7-Oxo-4,5,6,7-Tetrahydro-1*H*-Indole-2-Carboxamide (**15**)

This compound was obtained by reaction of **11** with 5-chloro-2-methoxyaniline after 16 h at 100 °C. White solid; yield 60%; mp: 280–281 °C; IR: ν_max_ = 3422 (NH), 3330 (NH), 1675 (CO), 1652 (CO) cm^−1^; ^1^H NMR (200 MHz, CDCl_3_) δ 2.09–2.21 (m, 2H, CH_2_), 2.56 (t, *J* = 6.0 Hz, 2H, CH_2_), 2.78 (t, *J* = 6.0 Hz, 2H, CH_2_), 3.92 (s, 3H, CH_3_), 6.53 (s, 1H, H-3), 6.80 (d, *J* = 8.8 Hz, 1H, Ar), 7.01 (dd, *J* = 8.8, 2.5 Hz, 1H, Ar), 8.33 (s, 1H, NH), 8.48 (d, *J* = 2.5 Hz, 1H, Ar), 10.08 (s, 1H, NH); ^13^C NMR (50 MHz, CDCl_3_) δ 23.1 (t), 24.9 (t), 38.1 (t), 56.2 (q), 108.2 (d), 110.8 (d), 119.8 (d), 123.6 (d), 126.3 (s), 128.0 (s), 130.1 (s), 130.7 (s), 136.3 (s), 146.4 (s), 157.8 (s), 189.5 (s). Anal calcd for C_16_H_15_ClN_2_O_3_: C, 60.29; H, 4.74; N, 8.79. Found: C, 60.14; H, 4.91; N, 8.94.

##### *N*-(5-Chloro-2-Methoxyphenyl)-1-Methyl-7-Oxo-4,5,6,7-Tetrahydro-1*H*-Indole-2-Carboxamide (**16**)

This compound was obtained by reaction of **12** with 5-chloro-2- methoxyaniline after 20 h at 100 °C. White solid; yield 60%; mp: 280–281 °C; IR: ν_max_ = 3422 (NH), 1680 (CO), 1658 (CO) cm^−1^; ^1^H NMR (200 MHz, CDCl_3_) δ 2.03–2.15 (m, 2H, CH_2_), 2.55 (t, *J* = 6.1 Hz, 2H, CH_2_), 2.77 (t, *J* = 6.1 Hz, 2H, CH_2_), 3.92 (s, 3H, CH_3_), 4.26 (s, 3H, CH_3_), 6.46 (s, 1H, H-3), 6.81 (d, *J* = 8.7 Hz, 1H, Ar), 7.03 (dd, *J* = 8.7, 2.5 Hz, 1H, Ar), 8.32 (s, 1H, NH), 8.49 (d, *J* = 2.5 Hz, 1H, Ar); ^13^C NMR (50 MHz, CDCl_3_) δ 23.7 (t), 25.6 (t), 34.8 (q), 40.0 (t), 56.2 (q), 109.4 (d), 110.7 (d), 119.5 (d), 123.4 (d), 126.3 (s), 128.3 (s), 130.2 (s), 131.9 (s), 135.4 (s), 146.4 (s), 159.1 (s), 190.5 (s). Anal calcd for C_17_H_17_ClN_2_O_3_: C, 61.36; H, 5.15; N, 8.42. Found: C, 61.22; H, 4.97; N, 8.63.

##### *N*-(5-Chloro-2-Methoxyphenyl)-8-Oxo-1,4,5,6,7,8-Hexahydrocyclohepta[*b*]Pyrrole-2-Carboxamide (**17**)

This compound was obtained by reaction of **13** with 5-chloro-2- methoxyaniline after 3 h at room temperature. White solid; yield 67%; mp: 280–281 °C; IR: ν_max_ = 3588 (NH), 3428 (NH), 1684 (CO), 1672 (CO) cm^−1^; ^1^H NMR (200 MHz, DMSO-d_6_) δ 1.81–1.84 (m, 4H, 2 × CH_2_), 2.66 (t, *J* = 5.7 Hz, 2H, CH_2_), 2.83 (t, *J* = 5.7 Hz, 2H, CH_2_), 3.88 (s, 3H, CH_3_), 6.78 (s, 1H, H-3), 7.11 (d, *J* = 8.9 Hz, 1H, Ar), 7.21 (dd, *J* = 8.9, 2.5 Hz, 1H, Ar), 7.91 (dd, *J* = 2.5 Hz, 1H, Ar), 9.68 (s, 1H, NH), 12.15 (s, 1H, NH); ^13^C NMR (50 MHz, DMSO-d_6_) δ 21.8 (t), 25.6 (t), 26.4 (t), 41.4 (t), 56.1 (q), 112.7 (d), 115.3 (d), 123.1 (d), 123.6 (s), 124.6 (d), 127.5 (s), 129.7 (s), 131.8 (s), 131.9 (s), 149.5 (s), 157.9 (s), 192.3 (s). Anal calcd for C_17_H_17_ClN_2_O_3_: C, 61.36; H, 5.15; N, 8.42. Found: C, 61.22; H, 5.31; N, 8.55.

##### *N*-Tert-Butyl-8-Oxo-1,4,5,6,7,8-Hexahydrocyclohepta[*b*]Pyrrole-2-Carboxamide (**18**)

This compound was obtained by reaction of **13** with tert-butylamine after 16 h at 100 °C. Light brown solid; yield 72%; mp: 280–281 °C; IR: ν_max_ = 3433 (NH), 3324 (NH), 1665 (CO), 1651 (CO) cm^−1^; ^1^H NMR (200 MHz, CDCl_3_) δ 1.44 (s, 9H, 3 × CH_3_), 1.89–1.98 (m, 4H, 2 × CH_2_), 2.70 (t, *J* = 5.4 Hz, 2H, CH_2_), 2.84 (t, *J* = 5.4 Hz, 2H, CH_2_), 5.92 (s, 1H, NH), 6.30 (s, 1H, H-3), 9.88 (s, 1H, NH); ^13^C NMR (50 MHz, CDCl_3_) δ 22.6 (t), 26.5 (t), 28.0 (t), 28.9 (3 × q), 42.2 (t), 51.8 (s), 110.1 (d), 130.7 (s), 130.8 (s), 132.4 (s), 159.5 (s), 192.7 (s). Anal calcd for C_14_H_20_N_2_O_2_: C, 67.71; H, 8.12; N, 11.28. Found: C, 67.89; H, 7.98; N, 11.12.

##### *N*-(5-Chloro-2-Methoxyphenyl)-1-Methyl-8-Oxo-1,4,5,6,7,8-Hexahydrocyclohepta[*b*]Pyrrole-2-Carboxamide (**19**)

This compound was obtained by reaction of **14** with 5-chloro-2-methoxyaniline after 5 h at room temperature. Light brown solid; yield 67%; mp: 280–281 °C; IR: ν_max_ = 3421 (NH), 1680 (CO), 1646 (CO) cm^−1^; ^1^H NMR (200 MHz, DMSO-d_6_) δ 1.74–1.77 (m, 4H, 2 × CH_2_), 2.64 (t, *J* = 5.8 Hz, 2H, CH_2_), 2.79 (t, *J* = 5.8 Hz, 2H, CH_2_), 3.85 (s, 3H, CH_3_), 4.02 (s, 3H, CH_3_), 6.79 (s, 1H, H-3), 7.11 (d, *J* = 8.9 Hz, 1H, Ar), 7.23 (dd, *J* = 8.9, 2.6 Hz, 1H, Ar), 7.88 (dd, *J* = 2.6 Hz, 1H, Ar), 9.32 (s, 1H, NH); ^13^C NMR (50 MHz, DMSO-d_6_) δ 20.9 (t), 24.5 (t), 25.1 (t), 34.5 (q), 41.4 (t), 56.1 (q), 112.8 (d), 113.2 (d), 122.7 (d), 123.6 (s), 124.8 (d), 127.7 (s), 130.5 (s), 132.4 (s), 133.0 (s), 149.7 (s), 159.3 (s), 193.7 (s). Anal calcd for C_18_H_19_ClN_2_O_3_: C, 62.34; H, 5.52; N, 8.08. Found: C, 62.58; H, 5.34; N, 7.88.

##### *N*-Tert-Butyl-1-Methyl-8-Oxo-1,4,5,6,7,8-Hexahydrocyclohepta[*b*]Pyrrole-2-Carboxamide (**20**)

This compound was obtained by reaction of **14** with tert-butylamine after 16 h at 50 °C. White solid; yield 67%; mp: 280–281 °C; IR: ν_max_ = 3336 (NH), 1663 (CO), 1635 (CO) cm^−1^; ^1^H NMR (200 MHz, DMSO-d_6_) δ 1.34 (s, 9H, 3 × CH_3_), 1.63–1.80 (m, 4H, 2 × CH_2_), 2.51–2.80 (m, 4H, 2 × CH_2_), 3.93 (s, 3H, CH_3_), 6.47 (s, 1H, H-3), 7.75 (s, 1H, NH); ^13^C NMR (50 MHz, DMSO-d_6_) δ 20.9 (t), 24.4 (t), 25.1 (t), 28.5 (3 × q), 34.3 (q), 41.3 (t), 50.9 (s), 112.1 (d), 130.9 (s), 132.9 (s), 133.0 (s), 160.9 (s), 193.2 (s). Anal calcd for C_15_H_22_N_2_O_2_: C, 68.67; H, 8.45; N, 10.68. Found: C, 68.81; H, 8.29; N, 10.53.

##### General Procedure for the Synthesis of α-Bromo-Ketones (**21–36**)

Method A. To a suspension of CuBr_2_ (5.4 mmol) in anhydrous ethyl acetate (30 mL), the suitable ketones (3 mmol) was added and the reaction mixture was heated at reflux for 2 h. After cooling, the reaction mixture was filtered through a celite pad and the filtrate was evaporated under reduced pressure. In the case of compound **23**, the residue was purified by column chromatography (dichloromethane).

Method B. To a solution of the suitable ketones (5 mmol) in anhydrous THF (10 mL), a solution of pyridine hydrobromide perbromide (5 mmol) in anhydrous THF (5 mL), was added and the reaction mixture was stirred at room temperature for 16 h. The solid formed was filtered through a celite pad and the filtrate was evaporated under reduced pressure. The residue was dissolved in dichloromethane (10 mL), washed with a solution of 5% aq. NaHCO_3_ (10 mL), dried over Na_2_SO_4_ and evaporated under reduced pressure. In the case of compounds **24**,**33**–**36**, the residue was purified by chromatography (dichloromethane).

##### 6-Bromo-1-(Phenylsulfonyl)-1,4,5,6-Tetrahydro-7*H*-Indol-7-One (**21**)

This compound was obtained by reaction of **4** (method A) and used in the next step without further purification.

##### 7-Bromo-1-(Phenylsulfonyl)-4,5,6,7-Tetrahydrocyclohepta[*b*]Pyrrol-8(1*H*)-One (**23**)

This compound was obtained by reaction of **7** using method A. Colorless oil; yield 82%; IR: ν_max_ = 1622 (CO) cm^−1^; ^1^H NMR (200 MHz, CDCl_3_) δ 1.78–2.35 (m, 4H, 2 × CH_2_), 2.63–3.00 (m, 2H, CH_2_), 4.64–4.75 (m, 1H, CH), 6.16 (d, *J* = 2.8 Hz, 1H, H-3), 7.48–7.63 (m, 3H, H-3′, H-4′ and H-5′), 7.70 (d, *J* = 2.8 Hz, 1H, H-2), 7.94 – 8.04 (m, 2H, H-2′ and H-6′); ^13^C NMR (50 MHz, CDCl_3_) δ 22.80 (t), 28.03 (t), 32.24 (t), 55.22 (d), 113.08 (d), 128.04 (2 × d), 128.78 (2 × d), 129.00 (s), 129.67 (d), 133.63 (d), 137.46 (s), 139.39 (s), 184.83 (s). Anal. Calcd. for C_15_H_14_BrNO_3_S: C, 48.92; H, 3.83; N, 3.80. Found: 49.03; H, 3.90; N, 4.00.

##### 1-(Benzoyl)-7-Bromo-4,5,6,7-Tetrahydrocyclohepta[*b*]Pyrrol-8(1*H*)-One (**24**)

This compound was obtained by reaction of **10b** using method B. Yellow oil; yield: 60%; IR: ν_max_ = 1711 (CO) 1636 (CO) cm^−1^; ^1^H NMR (200 MHz, CDCl_3_) δ 1.93–2.02 (m, 2H, CH_2_), 2.33–2.42 (m, 2H, CH_2_), 2.82–3.09 (m, 2H, CH_2_), 4.63 – 4.70 (m, 1H, CH), 6.12 (d, *J* = 2.6 Hz, 1H, H-3), 7.24 (d, *J* = 2.6 Hz, 1H, H-2), 7.41 – 7.76 (m, 5H, Ar); ^13^C NMR (50 MHz, CDCl_3_) δ 22.81 (t), 28.53 (t), 32.32 (t), 55.27 (d), 112.46 (d), 128.61 (d), 129.77 (2 × d), 129.92 (2 × d), 130.14 (s), 133.02 (s), 133.38 (d), 136.29 (s), 168.58 (s), 184.62 (s). Anal. Calcd. for C_16_H_14_BrNO_2_: C, 57.85; H, 4.25; N, 4.22. Found: 57.95; H, 4.32; N, 4.01.

##### Ethyl 7-Bromo-8-Oxo-1,4,5,6,7,8-Hexahydrocyclohepta[*b*]Pyrrole-2-Carboxylate (**25**)

This compound was obtained by reaction of **9** using method A and used in the next step without further purification.

##### Ethyl 7-Bromo-1-Methyl-8-Oxo-1,4,5,6,7,8-Hexahydrocyclohepta[*b*]Pyrrole-2-Carboxylate (**26**)

This compound was obtained by reaction of **10c** (method A) and used in the next step without further purification.

##### Ethyl 7-Bromo-1-(4-Methoxybenzyl)-8-Oxo-1,4,5,6,7,8-Hexahydrocyclohepta[*b*]Pyrrole-2-Carboxylate (**27**)

This compound was obtained by reaction of **10d** (method A) and used in the next step without further purification.

##### Ethyl 7-Bromo-1-(3,5-Dimethoxybenzyl)-8-Oxo-1,4,5,6,7,8-Hexahydrocyclohepta[*b*]Pyrrole-2-Carboxylate (**28**)

This compound was obtained by reaction of **10e** (method A) and used in the next step without further purification.

##### Ethyl 7-Bromo-1-(3,4,5-Trimethoxybenzyl)-8-Oxo-1,4,5,6,7,8-Hexahydrocyclohepta[*b*] Pyrrole-2-Carboxylate (**29**)

This compound was obtained by reaction of **10f** using method A and used in the next step without further purification.

##### Ethyl 7-Bromo-1-(2-Bromo-3,5-Dimethoxybenzyl)-8-Oxo-1,4,5,6,7,8-Hexahydrocyclohepta[*b*]Pyrrole-2-Carboxylate (**30**)

This compound was obtained by reaction of **10e** (method A) and used in the next step without further purification.

##### 6-Bromo-*N*-(5-Chloro-2-Methoxyphenyl)-7-Oxo-4,5,6,7-Tetrahydro-1*H*-Indole-2-Carboxamide (**31**)

This compound was obtained by reaction of **15** (method B) and used in the next step without further purification.

##### 6-Bromo-*N*-(5-Chloro-2-Methoxyphenyl)-1-Methyl-7-Oxo-4,5,6,7-Tetrahydro-1*H*-Indole-2-Carboxamide (**32**)

This compound was obtained by reaction of **16** (method B) and used in the next step without further purification.

##### 7-Bromo-*N*-(5-Chloro-2-Methoxyphenyl)-8-Oxo-1,4,5,6,7,8-Hexahydrocyclohepta[*b*]Pyrrole-2-Carboxamide (**33**)

This compound was obtained by reaction of **17** using method B. Brown solid; yield 92%; mp: 280–281 °C; IR: ν_max_ = 3422 (NH), 3273 (NH), 1669 (CO), 1635 (CO) cm^−1^; ^1^H NMR (200 MHz, DMSO-d_6_) δ 1.92–2.45 (m, 4H, 2 × CH_2_), 2.78–3.00 (m, 2H, CH_2_), 3.88 (s, 3H, CH_3_), 5.12–5.17 (m, 1H, CH), 6.79 (s, 1H, H-3), 7.12 (d, J = 8.9 Hz, 1H, Ar), 7.22 (dd, *J* = 8.9, 2.5 Hz, 1H, Ar), 7.90 (d, *J* = 2.5 Hz, 1H, Ar), 9.71 (s, 1H, NH), 12.28 (s, 1H, NH); ^13^C NMR (50 MHz, DMSO-d_6_) δ 23.1 (t), 27.1 (t), 32.0 (t), 56.1 (q), 56.7 (d), 112.8 (d), 115.6 (d), 123.2 (d), 123.6 (s), 124.7 (d), 127.4 (s), 128.9 (s), 131.3 (s), 131.6 (s), 149.6 (s), 157.5 (s), 185.4 (s). Anal calcd for C_17_H_16_BrClN_2_O_3_: C, 49.60; H, 3.92; N, 6.80. Found: C, 49.79; H, 4.05; N, 6.67.

##### 7-Bromo-*N*-Tert-Butyl-8-Oxo-1,4,5,6,7,8-Hexahydrocyclohepta[*b*]Pyrrole-2-Carboxamide (**34**)

This compound was obtained by reaction of **18** using method B. Light brown solid; yield 97%; mp: 280–281 °C; IR: ν_max_ = 3565 (NH), 3445 (NH), 1658 (CO), 1645 (CO) cm^−1^; ^1^H NMR (200 MHz, CDCl_3_) δ 1.44 (s, 9H, 3 × CH_3_), 1.55–2.05 (m, 2H, CH_2_), 2.19–2.39 (m, 2H, CH_2_), 2.77–3.07 (m, 2H, CH_2_), 4.83–4.87 (m, 1H, CH), 6.20 (s, 1H, NH), 6.39 (s, 1H, H-3), 10.09 (s, 1H, NH); ^13^C NMR (50 MHz, CDCl_3_) δ 23.2 (t), 28.2 (t), 28.8 (3 × q), 32.4 (t), 52.0 (s), 54.7 (d), 111.0 (d), 128.1 (s), 132.3 (s), 132.4 (s), 159.3 (s), 185.8 (s). Anal calcd for C_14_H_19_BrN_2_O_2_: C, 51.39; H, 5.85; N, 8.56. Found: C, 51.23; H, 5.99; N, 8.39.

##### 7-Bromo-*N*-(5-Chloro-2-Methoxyphenyl)-1-Methyl-8-Oxo-1,4,5,6,7,8-Hexahydrocyclohepta[*b*]Pyrrole-2-Carboxamide (**35**)

This compound was obtained by reaction of **19** using method B. Light brown solid; yield 65%; mp: 280–281 °C; IR: ν_max_ = 3378 (NH), 1661 (CO), 1648 (CO) cm^−1^; ^1^H NMR (200 MHz, DMSO-d_6_) δ 1.76–1.96 (m, 2H, CH_2_), 2.11–2.46 (m, 2H, CH_2_), 2.85 (t, *J* = 4.2 Hz, 2H, CH_2_), 3.85 (s, 3H, CH_3_), 3.91 (s, 3H, CH_3_), 5.11–5.17 (m, 1H, CH), 6.78 (s, 1H, H-3), 7.12 (d, *J* = 8.8 Hz, 1H, Ar), 7.24 (dd, *J* = 8.8, 2.5 Hz, 1H, Ar), 7.86 (d, *J* = 2.5 Hz, 1H, Ar), 9.41 (s, 1H, NH); ^13^C NMR (50 MHz, DMSO-d_6_) δ 26.0 (t), 31.3 (t), 33.2 (t), 34.6 (q), 47.5 (d), 56.1 (q), 111.8 (s), 112.8 (d), 113.3 (d), 123.5 (s), 125.0 (d), 127.2 (d), 130.9 (s), 142.3 (s), 146.3 (s), 159.3 (s), 163.6 (s), 189.6 (s). Anal calcd for C_18_H_18_BrClN_2_O_3_: C, 50.78; H, 4.26; N, 8.33. Found: C, 50.90; H, 4.14; N, 8.21.

##### 7-Bromo-*N*-Tert-Butyl-1-Methyl-8-Oxo-1,4,5,6,7,8-Hexahydrocyclohepta[*b*]Pyrrole-2-Carboxamide (**36**)

This compound was obtained by reaction of **20** using method B. Brown solid; yield 65%; mp: 280–281 °C; IR: ν_max_ = 3433 (NH), 1669 (CO), 1652 (CO) cm^−1^; ^1^H NMR (200 MHz, DMSO-d_6_) δ 1.35 (s, 9H, 3 × CH_3_), 1.85–2.49 (m, 4H, 2 × CH_2_), 2.78–2.84 (m, 2H, CH_2_), 3.82 (s, 3H, CH_3_), 5.06–5.12 (m, 1H, CH), 6.46 (s, 1H, H-3), 7.83 (s, 1H, NH); ^13^C NMR (50 MHz, DMSO-d_6_) δ 23.6 (t), 26.4 (t), 28.4 (3 × q), 32.3 (t), 34.3 (q), 41.4 (s), 58.4 (d), 112.3 (d), 129.0 (s), 132.6 (s), 137.0 (s), 166.1 (s), 187.6 (s). Anal calcd for C_15_H_21_BrN_2_O_2_: C, 52.80; H, 6.20; N, 8.21. Found: C, 52.97; H, 6.03; N, 8.39.

##### Synthesis of 7-Bromo-4,5,6,7-Tetrahydrocyclohepta[*b*]Pyrrol-8(1*H*)-One (**22**)

To a solution of **24** (5 mmol) in methanol (100 mL), a solution of 5 M aq. NaOH (1.1 mL, 5.5 mmol) was added and the reaction mixture was stirred at room temperature for 4 h. The solution was concentrated, cooled at 0 °C and acidified using HCl 6M. The aqueous phase was extracted with dichloromethane (3 × 20 mL) and the organic phase was washed with a solution of 5% aq. NaHCO_3_. Then the organic phase was dried over Na_2_SO_4_ and evaporated under reduced pressure. The residue was purified by column chromatography (dichloromethane). Pale brown solid; yield: 72 %; mp: 99.9–100.5 °C; IR: ν_max_ = 3425 (NH), 1622 (CO) cm^−1^; ^1^H NMR (200 MHz, CDCl_3_) δ 1.94–2.03 (m, 2H, CH_2_), 2.33–2.39 (m, 2H, CH_2_), 2.85–3.08 (m, 2H, CH_2_), 4.83–4.90 (m, 1H, CH), 6.10 (d, *J* = 2.4 Hz, 1H, H-3), 7.03 (d, *J* = 2.4 Hz, 1H, H-2), 9.45 (s, 1H, NH); ^13^C NMR (50 MHz, CDCl_3_) δ 23.3 (t), 28.5 (t), 32.8 (t), 55.0 (d), 112.2 (d), 126.3 (d), 127.0 (s), 133.0 (s), 185.2 (s). Anal. Calcd. for C_9_H_10_BrNO: C, 47.39; H, 4.42; N, 6.14. Found: 47.50; H, 4.27; N, 6.38.

##### General Procedure for the Synthesis of [1,3]Thiazole Derivatives (**37**–**39**)

To a solution of the suitable α-bromo derivative **21**,**31**,**32** (0.5 mmol) in anhydrous DMF (5 mL), Na_2_CO_3_ (1 mmol) and the proper thiourea (1 mmol) were added and the mixture was stirred at room temperature for 16 h. The reaction was poured onto ice and brine. In the case of formation of a precipitate, the solid was collected by filtration, in the absence of precipitate, the aqueous solution was extracted with dichloromethane (3 × 40 mL). The organic phase was dried over Na_2_SO_4_ and the solvent evaporated under reduced pressure. The crude product was purified by column chromatography or crystallization.

##### 8-(Phenylsulfonyl)-5,8-Dihydro-4*H*-[1,3]Thiazolo[5,4-*g*]Indol-2-Amine (**37**)

This compound was obtained by reaction of **21** with thiourea and was purified by crystallization from ethanol. Brown solid; yield 90%; mp: 79.2–80.1 °C (EtOH); IR: ν_max_ = 3415–3275 (NH_2_) cm^−1^; ^1^H NMR (200 MHz, DMSO-d_6_) δ 2.65–2.75 (m, 4H, 2 × CH_2_), 6.26 (d, *J* = 3.2 Hz, 1H, H-7), 6.84 (s, 2H, NH_2_), 7.31 (d, *J* = 3.2 Hz, 1H, H-8), 7.53–7.75 (m, 3H, H-3′, H-4′ and H-5′), 8.24–8.31 (m, 2H, H-2′ and H-6′); ^13^C NMR (500 MHz, DMSO-d_6_) δ 21.52 (t), 22.47 (t), 110.87 (d), 114.78 (s), 120.99 (d), 123.55 (s), 125.39 (s), 128.78 (2 × d), 129.08 (2 × d), 139.97 (d), 137.55 (s), 138.19 (s), 164.89 (s); Anal. Calcd. for C_15_H_13_N_3_O_2_S_2_: C, 54.36; H, 3.95; N, 12.68. Found: C, 54.12; H, 4.08; N, 12.79.

##### 2-Amino-*N*-(5-Chloro-2-Methoxyphenyl)-5,8-Dihydro-4*H*-[1,3]Thiazolo[5,4-*g*]Indole-7-Carboxamide (**38**)

This compound was obtained by reaction of **31** with thiourea and was purified by crystallization from methanol. Yellow solid; mp: 214–215 °C; yield 70%; IR: ν_max_ = 3422 (NH), 3330 (NH), 3205–3134 (NH_2_), 1669 (CO) cm^−1^; ^1^H NMR (200 MHz, DMSO-d_6_) δ 2.71–2.90 (m, 4H, 2 × CH_2_), 3.89 (s, 3H, CH_3_), 6.77 (s, 1H, H-6), 6.92 (s, 2H, NH_2_), 7.10–7.19 (m, 2H, Ar), 8.04 (d, *J* = 2.2 Hz, 1H, Ar), 9.12 (s, 1H, NH), 11.90 (s, 1H, NH); ^13^C NMR (50 MHz, DMSO-d_6_) δ 21.7 (t), 22.3 (t), 56.1 (q), 112.4 (d), 113.4 (d), 116.0 (s), 117.9 (s), 121.7 (s), 121.7 (d), 123.2 (d), 123.7 (s), 128.6 (s), 129.4 (s), 139.1 (s), 148.6 (s), 158.2 (s), 167.1 (s). Anal calcd for C_17_H_15_ClN_4_O_2_S: C, 54.47; H, 4.03; N, 14.95. Found: C, 54.63; H, 3.85; N, 15.08.

##### 2-Amino-*N*-(5-Chloro-2-Methoxyphenyl)-8-Methyl-5,8-Dihydro-4*H*-[1,3]Thiazolo[5,4-*g*]Indole-7-Carboxamide (**39**)

This compound was obtained by reaction of **32** with thiourea and was purified by column chromatography (dichloromethane). Grey solid; yield 93%; mp: 173–174 °C; IR: ν_max_ = 3426 (NH), 3324–3206 (NH_2_), 1664 (CO) cm^−1^; ^1^H NMR (200 MHz, DMSO-d_6_) δ 2.68–2.82 (m, 4H, 2 × CH_2_), 3.86 (s, 3H, CH_3_), 4.23 (s, 3H, CH_3_), 6.86 (s, 1H, H-6), 6.99–7.22 (m, 4H, Ar and NH_2_), 8.03 (s, 1H, Ar), 8.74 (s, 1H, NH); ^13^C NMR (50 MHz, DMSO-d_6_) δ 22.0 (t), 22.1 (t), 34.4 (q), 56.2 (q), 112.2 (d), 112.3 (d), 117.0 (s), 117.3 (s), 120.9 (d), 122.8 (s), 123.1 (d), 123.8 (s), 128.7 (s), 131.4 (s), 138.8 (s), 148.5 (s), 159.3 (s), 166.6 (s). Anal calcd for C_18_H_17_ClN_4_O_2_S: C, 55.59; H, 4.41; N, 14.41. Found: C, 55.72; H, 4.61; N, 14.29.

##### General Procedure for the Synthesis of *N*-Substituted[1,3]Thiazole Derivatives (**40**–**45**)

To a solution of the suitable 2-amine-thiazole **37**–**39** (0.46 mmol) in anhydrous 1,4-dioxane (3 mL), Et_3_N (0.07 mL, 0.51 mmol) and the suitable acyl chloride (0.69 mmol) were added and the reaction mixture was stirred at room temperature for 16 h. Then the reaction mixture was poured onto crushed ice. The precipitate was collected by filtration, dried and purified by column chromatography (dichloromethane).

##### 2-Methyl-*N*-[8-(Phenylsulfonyl)-5,8-Dihydro-4*H*-[1,3]Thiazolo[5,4-*g*]Indol-2-yl]Propanamide (**40**)

This compound was obtained by reaction of **37** with isobutyryl chloride. White solid; mp: 186–187 °C; yield 60%; IR: ν_max_ = 3313 (NH), 1680 (CO) cm^−1^; ^1^H NMR (200 MHz, CDCl_3_) δ 1.07 (s, 3H, CH_3_), 1.10 (s, 3H, CH_3_), 2.35–2.56 (m, 1H, CH), 2.67–2.89 (m, 4H, 2 × CH_2_), 6.23 (m, *J* = 3.3 Hz, 1H, Ar), 7.33–7.53 (m, 4H, Ar), 8.09–8–14 (m, 2H, Ar), 10.10 (s, 1H, NH); ^13^C NMR (50 MHz, CDCl_3_) δ 19.2 (2 × q), 22.1 (t), 23.0 (t), 35.3 (d), 111.5 (d), 122.5 (d), 123.0 (s), 125.4 (s), 125.7 (s), 128.1 (2 × d), 128.8 (2 × d), 133.8 (d), 137.1 (s), 138.3 (s), 155.2 (s), 175.1 (s). Anal calcd for C_19_H_19_N_3_O_3_S_2_: C, 56.84; H, 4.77; N, 10.47. Found: C, 56.72; H, 4.89; N, 10.63.

##### *N*-(5-Chloro-2-Methoxyphenyl)-2-[(2-Methylpropanoyl)Amino]-5,8-Dihydro-4*H*-[1,3]Thiazolo[5,4-*g*]Indole-7-Carboxamide (**41**)

This compound was obtained by reaction of **38** with isobutyryl chloride. White solid; mp: 255–256 °C; yield 68%; IR: ν_max_ = 3610 (NH), 3582 (NH), 3561 (NH), 1700 (CO), 1684 (CO) cm^−1^; ^1^H NMR (200 MHz, DMSO-d_6_) δ 1.12 (s, 3H, CH_3_), 1.15 (s, 3H, CH_3_), 2.75–2.85 (m, 3H, CH_2_ and CH), 2.94 (t, *J* = 7.4 Hz, 2H, CH_2_), 3.89 (s, 3H, CH_3_), 6.87 (s, 1H, H-6), 7.07–7.18 (m, 2H, Ar), 8.04 (d, *J* = 2.2 Hz, 1H, Ar), 9.07 (s, 1H, NH), 11.81 (s, 1H, NH), 11.93 (s, 1H, NH); ^13^C NMR (50 MHz, DMSO-d_6_) δ 19.2 (2 × q), 21.6 (t), 22.0 (t), 33.6 (d), 56.1 (q), 112.5 (d), 112.7 (d), 119.0 (s), 121.7 (d), 122.4 (s), 123.4 (d), 123.7 (s), 124.2 (s), 128.5 (s), 128.8 (s), 138.3 (s), 148.7 (s), 156.0 (s), 158.4 (s), 175.2 (s). Anal calcd for C_21_H_21_ClN_4_O_3_S: C, 56.69; H, 4.76; N, 12.59. Found: C, 56.55; H, 4.49; N, 12.73.

##### *N*-(5-Chloro-2-Methoxyphenyl)-8-Methyl-2-[(2-Methylpropanoyl)Amino]-5,8-Dihydro-4*H*-[1,3]Thiazolo[5,4-*g*]Indole-7-Carboxamide (**42**)

This compound was obtained by reaction of **39** with isobutyryl chloride. White solid; mp: 240–241 °C; yield 81%; IR: ν_max_ = 3428 (NH), 3250 (NH), 1664 (CO), 1658 (CO) cm^−1^; ^1^H NMR (200 MHz, CDCl_3_) δ 1.31 (s, 3H, CH_3_), 1.34 (s, 3H, CH_3_), 2.66–2.99 (m, 5H, 2 × CH_2_ and CH), 3.91 (s, 3H, CH_3_), 4.35 (s, 3H, CH_3_), 6.59 (s, 1H, H-6), 6.79 (d, *J* = 8.7 Hz, 1H, Ar), 6.98 (dd, *J* = 8.7, 2.4 Hz, 1H, Ar), 8.23 (s, 1H, NH), 8.49 (d, *J* = 2.4 Hz, 1H, Ar), 10.34 (s, 1H, NH); ^13^C NMR (50 MHz, CDCl_3_) δ 19.1 (2 × q), 22.2 (t), 22.7 (t), 35.0 (q), 35.7 (d), 56.1 (q), 110.6 (d), 110.8 (s), 111.0 (d), 119.2 (d), 119.3 (s), 122.5 (d), 123.9 (s), 125.3 (s), 126.3 (s), 129.0 (s), 129.6 (s), 146.3 (s), 156.5 (s), 159.4 (s), 174.6 (s). Anal calcd for C_22_H_23_ClN_4_O_3_S: C, 57.57; H, 5.05; N, 12.21. Found: C, 57.72; H, 4.88; N, 12.40.

##### 2,2-Dimethyl-*N*-[8-(Phenylsulfonyl)-5,8-Dihydro-4*H*-[1,3]Thiazolo[5,4-*g*]Indol-2-yl]Propanamide (**43**)

This compound was obtained by reaction of **37** with trimethylacetyl chloride. White solid; mp: 87–88 °C; yield 65%; IR: ν_max_ = 3502 (NH), 1675 (CO) cm^−1^; ^1^H NMR (200 MHz, CDCl_3_) δ 1.28 (s, 9H, 3 × CH_3_), 2.68 – 2.98 (m, 4H, 2 × CH_2_), 6.20–6.26 (m, 1H, Ar), 7.35–7.68 (m, 4H, Ar), 7.91–8.08 (m, 2H, Ar), 9.94 (s, 1H, NH); ^13^C NMR (50 MHz, CDCl_3_) δ 22.0 (t), 22.9 (t), 26.9 (3 × q), 40.5 (s), 110.5 (d), 112.0 (d), 122.7 (s), 123.0 (s), 126.4 (s), 127.6 (2 × d), 128.8 (2 × d), 133.7 (d), 134.0 (s), 138.8 (s), 149.5 (s), 176.2 (s). Anal calcd for C_20_H_21_N_3_O_3_S_2_: C, 57.81; H, 5.09; N, 10.11. Found: C, 57.97; H, 4.90; N, 10.23.

##### *N*-(5-Chloro-2-Methoxyphenyl)-2-[(2,2-Dimethylpropanoyl)Amino]-5,8-Dihydro-4*H*-[1,3]Thiazolo[5,4-*g*]Indole-7-Carboxamide (**44**)

This compound was obtained by reaction of **38** with trimethylacetyl chloride. White solid; yield 65%; mp: 155–156 °C; IR: ν_max_ = 3420 (NH), 3404 (NH), 3259 (NH), 1669 (CO), 1653 (CO) cm^−1^; ^1^H NMR (200 MHz, CDCl_3_) δ 1.33 (s, 9H, 3 × CH_3_), 2.90–3.00 (m, 4H, 2 × CH_2_), 3.91 (s, 3H, CH_3_), 6.58 (s, 1H, H-6), 6.79 (d, *J* = 8.7 Hz, 1H, Ar), 6.97 (dd, *J* = 8.7, 2.3 Hz, 1H, Ar), 8.18 (s, 1H, NH), 8.52 (d, *J* = 2.3 Hz, 1H, Ar), 9.17 (s, 1H, NH), 10.24 (s, 1H, NH); ^13^C NMR (50 MHz, CDCl_3_) δ 22.2 (t), 22.7 (t), 27.2 (3 × q), 39.2 (s), 56.1 (q), 109.5 (d), 110.6 (d), 119.6 (d), 122.6 (d), 123.9 (s), 124.3 (s), 126.2 (s), 128.8 (s), 129.3 (s), 138.1 (s), 146.3 (s), 150.5 (s), 156.8 (s), 158.8 (s), 176.2 (s). Anal calcd for C_22_H_23_ClN_4_O_3_S: C, 57.57; H, 5.05; N, 12.21. Found: C, 57.78; H, 5.19; N, 12.08.

##### *N*-(5-Chloro-2-Methoxyphenyl)-2-[(2,2-Dimethylpropanoyl)Amino]-8-Methyl-5,8-Dihydro-4*H*-[1,3]Thiazolo[5,4-*g*]Indole-7-Carboxamide (**45**)

This compound was obtained by reaction of **39** with trimethylacetyl chloride. Brown solid; mp: 213–214 °C; yield 78%; IR: ν_max_ = 3430 (NH), 3412 (NH), 1684 (CO), 1675 (CO) cm^−1^; ^1^H NMR (200 MHz, CDCl_3_) δ 1.36 (s, 9H, 3 × CH_3_), 2.78–3.00 (m, 4H, 2 × CH_2_), 3.90 (s, 3H, CH_3_), 4.35 (s, 3H, CH_3_), 6.59 (s, 1H, H-6), 6.78 (d, *J* = 8.7 Hz, 1H, Ar), 6.96 (dd, *J* = 8.7, 2.5 Hz, 1H, Ar), 8.23 (s, 1H, NH), 8.51 (d, *J* = 2.5 Hz, 1H, Ar), 8.96 (s, 1H, NH); ^13^C NMR (50 MHz, CDCl_3_) δ 22.4 (t), 22.8 (t), 27.3 (3 × q), 34.9 (q), 39.1 (s), 56.1 (q), 110.6 (d), 111.0 (d), 118.3 (s), 119.1 (d), 122.3 (d), 124.4 (s), 125.0 (s), 126.2 (s), 129.2 (s), 131.4 (s), 138.6 (s), 146.3 (s), 155.3 (s), 159.6 (s), 176.0 (s). Anal calcd for C_23_H_25_ClN_4_O_3_S: C, 58.40; H, 5.33; N, 11.85. Found: C, 58.56; H, 5.19; N, 11.98.

##### General Procedure for the Synthesis of [1,3]Thiazole Derivatives (**46**–**59**)

To a solution of the suitable bromo derivative **22**,**23**,**25**–**30**,**33**–**36** (0.5 mmol) in anhydrous DMF (5 mL), Na_2_CO_3_ (1 mmol) and the proper thiourea (1 mmol) were added and the mixture was stirred at room temperature for 16 h. The reaction was poured onto ice and brine. In the case of formation of a precipitate, the solid was collected by filtration, in the absence of precipitate, the aqueous solution was extracted with dichloromethane (3 × 40 mL). The organic phase was dried over Na_2_SO_4_ and the solvent evaporated under reduced pressure. The crude product was purified by column chromatography or crystallization.

##### 4,5,6,9-Tetrahydropyrrolo[3′,2′:6,7]Cyclohepta[1,2-*d*][1,3]Thiazol-2-Amine (**46**)

This compound was obtained by reaction of **22** with thiourea and was purified by crystallization from ethanol. Brown solid; yield 64%; mp: 66.4–67.2 °C; IR: ν_max_ = 3455 3416 (NH_2_), 3287 (NH) cm^−1^; ^1^H NMR (200 MHz, CDCl_3_) δ 2.02–2.09 (m, 2H, CH_2_), 2.87–2.94 (4H, m, 2 × CH_2_), 4.97 (s, 2H, NH_2_), 5.99 (d, *J* = 2.9 Hz, 1H, H-7), 6.58 (d, *J* = 2.9 Hz, 1H H-8), 9.27 (s, 1H, NH); ^13^C NMR (50 MHz, CDCl_3_) δ 24.31 (t), 27.57 (t), 28.52 (t), 110.28 (d), 116.18 (d), 117.02 (s), 120.53 (s), 124.79 (s), 139.16 (s), 144.61 (s). Anal. Calcd. for C_10_H_11_N_3_S: C, 58.51; H, 5.40; N, 20.47. Found: C, 58.65; H, 5.53; N, 20.30.

##### 9-(Phenylsulfonyl)-4,5,6,9-Tetrahydropyrrolo[3′,2′:6,7]Cyclohepta[1,2-*d*][1,3]Thiazol-2-Amine (**47**)

This compound was obtained by reaction of **23** with thiourea and was purified by column chromatography (dichloromethane: ethyl acetate 8:2). Yellow solid; yield 64 %, mp: 173–174 °C; IR: ν_max_ = 3252–3143 (NH_2_) cm^−1^; ^1^H NMR (200 MHz, CDCl_3_) δ 2.01–2.11 (m, 2H, CH_2_), 2.41 (t, *J* = 6.7 Hz, 2H, CH_2_), 2.45 (t, *J* = 6.7 Hz, 2H, CH_2_), 4.68 (s, 2H, NH_2_), 6.17 (d, *J* = 3.0 Hz, 1H, H-7), 7.35 (d, *J* = 3.0 Hz, 1H, H-8), 7.40–7.55 (m, 3H, H-3′, H-4′ and H-5′), 7.86 (d, *J* = 7.0 Hz, 2H, H-2′ and H-6′); ^13^C NMR (50 MHz, CDCl_3_) δ 24.0 (t), 25.0 (t), 31.8 (t), 113.5 (d), 123.2 (d), 125.3 (s), 126.7 (s), 127.2 (2 × d), 128.4 (2 × d), 130.5 (s), 133.0 (d), 138.3 (s), 140.1 (s), 162.7 (s). Anal. Calcd. for C_16_H_15_N_3_O_2_S_2_: C, 55.63; H, 4.38; N, 12.16. Found: C, 55.79; H, 4.22; N, 11.89.

##### Ethyl 2-Amino-4,5,6,9-Tetrahydropyrrolo[3′,2′:6,7]Cyclohepta[1,2-*d*][1,3]Thiazole-8-Carboxylate (**48**)

This compound was obtained by reaction of **25** with thiourea and was purified by column chromatography (dichloromethane: ethyl acetate 8:2). White solid; yield 62%; mp: 190.3–191.1 °C; IR: ν_max_ = 3370 (NH), 3202–3156 (NH_2_), 1692 (CO) cm^−1^; ^1^H NMR (200 MHz, DMSO-d_6_) δ 1.27 (t, *J* = 7.1 Hz, 3H, CH_3_), 1.85–1.99 (m, 2H, CH_2_), 2.75–2.85 (m, 4H, 2 × CH_2_), 4.22 (q, *J* = 7.1 Hz, 2H, CH_2_), 6.63 (s, 1H, H-7), 6.88 (s, 2H, NH_2_), 9.64 (s, 1H, NH); ^13^C NMR (50 MHz, DMSO-d_6_) δ 14.33 (q), 23.91 (t), 27.04 (t), 27.64 (t), 59.55 (t), 116.41 (d), 118.95 (s), 119.33 (s), 121.62 (s), 129.87 (s), 137.86 (s), 159.91 (s), 165.52 (s). Anal. Calcd. for C_13_H_15_N_3_O_2_S: C, 56.30; H, 5.45; N, 15.15. Found: C, 56.16; H, 5.57; N, 15.30.

##### Ethyl 2-Amino-9-Methyl-4,5,6,9-Tetrahydropyrrolo[3′,2′:6,7]Cyclohepta[1,2-*d*][1,3]Thiazole-8-Carboxylate (**49**)

This compound was obtained by reaction of **26** with thiourea and was purified by column chromatography (dichloromethane: ethyl acetate 8:2). Yellow solid; yield 60%, mp: 113–114 °C; IR: ν_max_ = 3255–3188 (NH_2_), 1690 (CO) cm^−1^; ^1^H NMR (200 MHz, CDCl_3_) δ 1.34 (t, *J* = 7.1 Hz, 3H, CH_3_), 2.05–2.09 (m, 2H, CH_2_), 2.61 (t, *J* = 6.4 Hz, 2H, CH_2_), 2.76 (t, *J* = 6.4 Hz, 2H, CH_2_), 4.16 (s, 3H, CH_3_), 4.27 (q, *J* = 7.1 Hz, 2H, CH_2_), 4.86 (s, 2H, NH_2_), 6.79 (s, 1H, H-7); ^13^C NMR (50 MHz, CDCl_3_) δ 14.5 (q), 25.7 (t), 25.8 (t), 29.9 (t), 35.2 (q), 59.6 (t), 117.3 (d), 121.8 (s), 124.2 (s), 126.2 (s), 132.7 (s), 139.0 (s), 161.5 (s), 163.3 (s). Anal. Calcd. for C_14_H_17_N_3_O_2_S: C, 57.71; H, 5.88; N, 14.42. Found: C, 57.93; H, 5.67; N, 14.28.

##### Ethyl 2-Amino-9-(4-Methoxybenzyl)-4,5,6,9-Tetrahydropyrrolo[3′,2′:6,7]Cyclohepta[1,2-*d*][1,3]Thiazole-8-Carboxylate (**50**)

This compound was obtained by reaction of **27** with thiourea and was purified by column chromatography (dichloromethane: ethyl acetate 8:2). Yellow solid; yield 69%, mp: 59 °C; IR: ν_max_ = 3253–3175 (NH_2_), 1715 (CO) cm^−1^; ^1^H NMR (200 MHz, CDCl_3_) δ 1.27 (t, *J* = 7.1 Hz, 3H, CH_3_), 2.05–2.12 (m, 2H, CH_2_), 2.63 (t, *J* = 6.5 Hz, 2H, CH_2_), 2.73 (t, *J* = 6.5 Hz, 2H, CH_2_), 3.73 (s, 3H, CH_3_), 4.17 (q, *J* = 7.1 Hz, 2H, CH_2_), 4.73 (s, 2H, NH_2_), 6.09 (s, 2H, CH_2_), 6.73 (d, *J* = 8.6 Hz, 2H, H-3′ and H-5′), 6.86 (s, 1H, H-7) 6.89 (d, *J* = 8.6 Hz, 2H, H-2′ and H-6′); ^13^C NMR (50 MHz, CDCl_3_) δ 14.4 (q), 25.5 (t), 25.7 (t), 30.2 (t), 48.6 (t), 55.1 (q), 59.6 (t), 113.5 (2 × d), 118.6 (d), 121.4 (s), 124.7 (s), 126.3 (s), 127.5 (2 × d), 132.5 (s), 132.6 (s), 139.1 (s), 158.1 (s), 161.1 (s), 163.4 (s). Anal. Calcd. for C_21_H_23_N_3_O_3_S: C, 63.45; H, 5.83; N, 10.57. Found: C, 63.31; H, 5.97; N, 10.44.

##### Ethyl 2-Amino-9-(3,5-Dimethoxybenzyl)-4,5,6,9-Tetrahydropyrrolo[3′,2′:6,7]Cyclohepta[1,2-*d*][1,3]Thiazole-8-Carboxylate (**51**)

This compound was obtained by reaction of **28** with thiourea and was purified by column chromatography (dichloromethane: ethyl acetate 8:2). Brown solid; yield 58%, mp: 140.6–141.0 °C; IR: ν_max_ = 3377–3253 (NH_2_), 1696 (CO) cm^−1^; ^1^H NMR (200 MHz, CDCl_3_) δ 1.27 (t, *J* = 7.1 Hz, 3H, CH_3_), 2.03–2.13 (m, 2H, CH_2_), 2.63 (t, *J* = 6.6 Hz, 2H, CH_2_), 2.73 (t, *J* = 6.6 Hz, 2H, CH_2_), 3.66 (s, 6H, 2 × CH_3_), 4.19 (q, *J* = 7.1 Hz, 2H, CH_2_), 4.77 (s, 2H, NH_2_), 6.08 (s, 2H, CH_2_), 6.14 (s, 2H, H-2′ and H-6′), 6.23 (s, 1H, H-4′), 6.87 (s, 1H, H-7); ^13^C NMR (50 MHz, CDCl_3_) δ 14.4 (q), 25.6 (t), 25.7 (t), 30.2 (t), 49.1 (t), 55.1 (2 × q), 59.6 (t), 98.6 (d), 104.1 (2 × d), 118.6 (d), 121.7 (s), 124.7 (s), 126.1 (s), 132.7 (s), 139.1 (s), 142.9 (s), 160.5 (2 × s), 161.1 (s), 163.5 (s). Anal. Calcd. for C_22_H_25_N_3_O_4_S: C, 61.81; H, 5.89; N, 9.83. Found: C, 61.68; H, 5.72; N, 9.99.

##### Ethyl 2-Amino-9-(3,4,5-Trimethoxybenzyl)-4,5,6,9-Tetrahydropyrrolo[3′,2′:6,7]Cyclohepta[1,2-*d*][1,3]Thiazole-8-Carboxylate (**52**)

This compound was obtained by reaction of **29** with thiourea and was purified by column chromatography (dichloromethane: ethyl acetate 8:2). Yellow solid; yield 92%, mp: 149.5–150.3 °C; IR: ν_max_ = 3408–3357 (NH_2_), 1701 (CO) cm^−1^; ^1^H NMR (200 MHz, CDCl_3_) δ 1.30 (t, *J* = 7.1 Hz, 3H, CH_3_), 2.03–2.11 (m, 2H, CH_2_), 2.63 (t, *J* = 6.4 Hz, 2H, CH_2_), 2.73 (t, *J* = 6.4 Hz, 2H, CH_2_), 3.70 (s, 6H, 2 × CH_3_), 3.76 (s, 3H, CH_3_), 4.22 (q, *J* = 7.1 Hz, 2H, CH_2_), 4.78 (s, 2H, NH_2_), 6.14 (s, 2H, CH_2_), 6.23 (s, 2H, H-2′ and H-6′), 6.86 (s, 1H, H-7); ^13^C NMR (50 MHz, CDCl_3_) δ 14.4 (q), 25.5 (t), 25.6 (t), 30.4 (t), 48.8 (t), 55.7 (2 × q), 59.7 (t), 60.7 (q), 103.6 (2 × d), 118.7 (d), 121.6 (s), 124.9 (s), 126.1 (s), 132.6 (s), 135.9 (s), 136.3 (s), 139.3 (s), 152.8 (2 × s), 161.2 (s), 163.6 (s). Anal. Calcd. for C_23_H_27_N_3_O_5_S: C, 60.38; H, 5.95; N, 9.18. Found: C, 60.61; H, 6.11; N, 9.02.

##### Ethyl 2-Amino-9-(2-Bromo-3,5-Dimethoxybenzyl)-4,5,6,9-Tetrahydropyrrolo[3′,2′:6,7]Cyclohepta[1,2-*d*][1,3]Thiazole-8-Carboxylate (**53**)

This compound was obtained by reaction of **30** with thiourea and was purified by column chromatography (dichloromethane: ethyl acetate 8:2). Brown solid; yield 53%, mp: 189.3–189.9 °C; IR: ν_max_ = 3334–3227 (NH_2_), 1695 (CO) cm^−1^; ^1^H NMR (200 MHz, CDCl_3_) δ 1.25 (t, *J* = 7.1 Hz, 3H, CH_3_), 2.00–2.09 (m, 2H, CH_2_), 2.69–2.84 (m, 4H, 2 × CH_2_), 3.56 (s, 3H, CH_3_), 3.84 (s, 3H, CH_3_), 4.18 (q, J = 7.1 Hz, 2H, CH_2_), 4.62 (s, 2H, NH_2_), 5.43 (s, 1H, H-4′), 6.21 (s, 2H, CH_2_), 6.28 (s, 1H, H-6′), 6.91 (s, 1H, H-7); ^13^C NMR (50 MHz, CDCl_3_) δ 14.3 (q), 26.6 (t), 26.7 (t), 28.4 (t), 51.1 (t), 55.1 (q), 56.2 (q), 59.7 (t), 97.2 (d), 101.3 (s), 103.2 (d), 118.7 (d), 121.9 (s), 124.6 (s), 126.0 (s), 132.4 (s), 138.3 (s), 142.4 (s), 156.1 (s), 159.7 (s), 160.9 (s), 162.9 (s). Anal. Calcd. for C_22_H_24_BrN_3_O_4_S: C, 52.18; H, 4.78; N, 8.30. Found: C, 51.93; H, 4.55; N, 8.57.

##### 2-Amino-*N*-(5-Chloro-2-Methoxyphenyl)-4,5,6,9-Tetrahydropyrrolo[3′,2′:6,7]Cyclohepta[1,2-*d*][1,3]Thiazole-8-Carboxamide (**54**)

This compound was obtained by reaction of **33** with thiourea and was purified by column chromatography (petroleum ether: ethyl acetate 7:3). Yellow solid; mp: 240–241 °C; yield 96%; IR: ν_max_ = 3422 (NH), 3307 (NH), 3239–3205 (NH_2_), 1658 (CO) cm^−1^; ^1^H NMR (200 MHz, DMSO-d_6_) δ 1.84–2.01 (m, 2H, CH_2_), 2.74–2.89 (m, 4H, 2 × CH_2_), 3.87 (s, 3H, CH_3_), 6.73–6.82 (m, 3H, H-7 and NH_2_), 7.06–7.17 (m, 2H, Ar), 7.92 (s, 1H, Ar), 9.29 (s, 1H, NH), 10.43 (s, 1H, NH); ^13^C NMR (50 MHz, DMSO-d_6_) δ 24.0 (t), 27.1 (t), 27.8 (t), 56.1 (q), 112.5 (d), 114.8 (d), 118.7 (s), 121.5 (s), 122.6 (d), 122.7 (s), 123.8 (d), 123.7 (s), 128.3 (s), 129.1 (s), 137.6 (s), 149.2 (s), 158.4 (s), 165.4 (s). Anal calcd for C_18_H_17_ClN_4_O_2_S: C, 55.59; H, 4.41; N, 14.41. Found: C, 55.44; H, 4.58; N, 14.26.

##### 2-Amino-*N*-Tert-Butyl-4,5,6,9-Tetrahydropyrrolo[3′,2′:6,7]Cyclohepta[1,2-*d*][1,3]Thiazole-8-Carboxamide (**55**)

This compound was obtained by reaction of **34** with thiourea and was purified by column chromatography (petroleum ether: ethyl acetate 7:3). Light yellow solid; mp: 166–167 °C; yield 74%; IR: ν_max_ = 3433–3378 (NH_2_), 3312 (NH), 3186 (NH), 1635 (CO) cm^−1^; ^1^H NMR (200 MHz, DMSO-d_6_) δ 1.35 (s, 9H, 3 × CH_3_), 1.85–1.96 (m, 2H, CH_2_), 2.72–2.85 (m, 4H, 2 × CH_2_), 6.54 (s, 1H, H-7), 6.71 (s, 2H, NH_2_), 7.56 (s, 1H, NH), 10.26 (s, 1H, NH); ^13^C NMR (50 MHz, DMSO-d_6_) δ 24.1 (t), 27.1 (t), 27.9 (t), 28.8 (3 × q), 50.4 (s), 113.4 (d), 117.1 (s), 120.5 (s), 124.6 (s), 127.2 (s), 138.0 (s), 159.8 (s), 165.1 (s). Anal calcd for C_15_H_20_N_4_OS: C, 59.18; H, 6.62; N, 18.41. Found: C, 59.05; H, 6.81; N, 18.28.

##### 2-Amino-*N*-(5-Chloro-2-Methoxyphenyl)-9-Methyl- 4,5,6,9-Tetrahydropyrrolo[3′,2′:6,7]Cyclohepta[1,2-*d*][1,3]Thiazole-8-Carboxamide (**56**)

This compound was obtained by reaction of **35** with thiourea and was purified by crystallization from diethyl ether. Yellow solid; yield 93%; mp: 181–182 °C; IR: ν_max_ = 3428 (NH), 3268–3182 (NH_2_), 1669 (CO) cm^−1^; ^1^H NMR (200 MHz, DMSO-d_6_) δ 1.99–2.10 (m, 2H, CH_2_), 2.63 (t, *J* = 6.4 Hz, 2H, CH_2_), 2.78 (t, *J* = 6.4 Hz, 2H, CH_2_), 3.92 (s, 3H, CH_3_), 4.13 (s, 3H, CH_3_), 6.89 (s, 1H, H-7), 7.12–7.30 (m, 4H, Ar and NH_2_), 8.06 (d, *J* = 1.8 Hz, 1H, Ar), 8.92 (s, 1H, NH); ^13^C NMR (50 MHz, DMSO-d_6_) δ 25.2 (t), 25.5 (t), 29.2 (t), 35.0 (q), 56.2 (q), 112.4 (d), 113.8 (d), 121.2 (d), 122.9 (s), 123.5 (d), 125.0 (s), 126.4 (s), 126.8 (s), 127.3 (s), 128.8 (s), 147.8 (s), 155.4 (s), 156.0 (s), 160.6 (s). Anal calcd for C_19_H_19_ClN_4_O_2_S: C, 56.64; H, 4.75; N, 13.91. Found: C, 56.42; H, 4.99; N, 14.09.

##### 2-Amino-*N*-Tert-Butyl-9-Methyl-4,5,6,9-Tetrahydropyrrolo[3′,2′:6,7]Cyclohepta[1,2-*d*][1,3]Thiazole-8-Carboxamide (**57**)

This compound was obtained by reaction of **36** with thiourea and was purified by crystallization from diethyl ether. Light yellow solid; yield 90%; mp: 228–229 °C; IR: ν_max_ = 3416–3302 (NH_2_), 3188 (NH), 1646 (CO) cm^−1^; ^1^H NMR (200 MHz, DMSO-d_6_) δ 1.34 (s, 9H, 3 × CH_3_), 1.87–2.00 (m, 2H, CH_2_), 2.52 (t, *J* = 6.5 Hz, 2H, CH_2_), 2.69 (t, *J* = 6.5 Hz, 2H, CH_2_), 4.01 (s, 3H, CH_3_), 6.52 (s, 1H, H-7), 6.83 (s, 2H, NH_2_), 7.25 (s, 1H, NH); ^13^C NMR (50 MHz, DMSO-d_6_) δ 25.3 (t), 26.0 (t), 28.8 (3 × q), 29.1 (t), 34.6 (q), 50.4 (s), 112.7 (d), 121.0 (s), 121.8 (s), 126.3 (s), 129.8 (s), 138.5 (s), 161.5 (s), 164.4 (s). Anal calcd for C_16_H_22_N_4_OS: C, 60.35; H, 6.96; N, 17.59. Found: C, 60.57; H, 7.11; N, 17.46.

##### *N-Tert*-Butyl-2-[(5-Chloro-2-Methoxyphenyl)Amino]-4,5,6,9-Tetrahydropyrrolo[3′,2′:6,7]Cyclohepta[1,2-*d*][1,3]Thiazole-8-Carboxamide (**58**)

This compound was obtained by reaction of **34** with 1-(5-chloro-2-methoxyphenyl)thiourea and was purified by column chromatography (petroleum ether: ethyl acetate 7:3). Orange solid; mp: 134–135 °C; yield 65%; IR: ν_max_ = 3438 (NH), 3405 (NH), 3291 (NH), 1710 (CO) cm^−1^; ^1^H NMR (200 MHz, CDCl_3_) δ 1.45 (s, 9H, 3 × CH_3_), 2.00–2.15 (m, 2H, CH_2_), 2.83–2.97 (m, 4H, 2 × CH_2_), 3.89 (s, 3H, CH_3_), 5.77 (s, 1H, NH), 6.32 (s, 1H, H-7), 6.77 (d, *J* = 8.6 Hz, 1H, Ar), 6.91 (dd, *J* = 8.6, 2.0 Hz, 1H, Ar), 7.26 (s, 1H, NH), 7.88 (d, *J* = 2.0 Hz, 1H, Ar), 9.83 (s, 1H, NH); ^13^C NMR (50 MHz, CDCl_3_) δ 24.2 (t), 27.6 (t), 28.5 (t), 29.2 (3 × q), 51.4 (s), 56.0 (q), 110.6 (d), 110.9 (d), 115.9 (d), 120.1 (s), 121.1 (d), 122.1 (s), 124.6 (s), 126.1 (s), 127.7 (s), 130.8 (s), 146.0 (s), 160.0 (s), 160.7 (s), 176.1 (s). Anal calcd for C_22_H_25_ClN_4_O_2_S: C, 59.38; H, 5.66; N, 12.59. Found: C, 59.53; H, 5.79; N, 12.42.

##### *N*-*tert*-Butyl-2-[(5-Chloro-2-Methoxyphenyl)Amino]-9-Methyl-4,5,6,9-Tetrahydropyrrolo[3′,2′:6,7]Cyclohepta[1,2-*d*][1,3]Thiazole-8-Carboxamide (**59**)

This compound was obtained by reaction of **36** with 1-(5-chloro-2-methoxyphenyl)thiourea and was purified by column chromatography (petroleum ether: ethyl acetate 9:1). Light brown solid; mp: 163–164 °C; yield 85%; IR: ν_max_ = 3588 (NH), 3559 (NH), 1703 (CO) cm^−1^; ^1^H NMR (200 MHz, CDCl_3_) δ 1.45 (s, 9H, 3 × CH_3_), 2.02–2.15 (m, 2H, CH_2_), 2.66 (t, *J* = 6.5 Hz, 2H, CH_2_), 2.85 (t, *J* = 6.5 Hz, 2H, CH_2_), 3.90 (s, 3H, CH_3_), 4.26 (s, 3H, CH_3_), 5.74 (s, 1H, NH), 6.30 (s, 1H, H-7), 6.77 (d, J = 8.6 Hz, 1H, Ar), 6.89 (dd, *J* = 8.6, 2.4 Hz, 1H, Ar), 7.48 (s, 1H, NH), 8.39 (d, *J* = 2.4 Hz, 1H, Ar); ^13^C NMR (50 MHz, CDCl_3_) δ 25.8 (t), 26.3 (t), 29.1 (3 × q), 29.4 (t), 35.5 (q), 51.3 (s), 56.0 (q), 110.5 (d), 111.2 (d), 116.5 (d), 120.5 (d), 120.6 (s), 123.5 (s), 124.2 (s), 126.3 (s), 127.1 (s), 130.8 (s), 139.6 (s), 145.4 (s), 158.5 (s), 162.0 (s). Anal calcd for C_23_H_27_ClN_4_O_2_S: C, 60.18; H, 5.93; N, 12.21. Found: C, 59.95; H, 6.08; N, 12.45.

##### General Procedure for the Synthesis of *N*-Acetyl-[1,3]Thiazole Derivatives (**60**–**65**)

To a solution of the suitable 2-amine-thiazole **47**,**49**–**53** (0.5 mmol) in anhydrous dichloromethane (5 mL), DIPEA (0.1 mL, 0.6 mmol) and acetyl chloride (0,04 mL, 0.55 mmol) were added at 0 °C, and the reaction mixture was stirred at room temperature for 24 h. Water (3 mL) was added and the organic phase was separated and the aqueous phase was extracted with dichloromethane (2 × 5 mL). The organic phase was dried over Na_2_SO_4_ and the solvent evaporated under reduced pressure. The residue was purified by column chromatography (dichloromethane: ethyl acetate 95:5).

##### *N*-[9-(Phenylsulfonyl)-4,5,6,9-Tetrahydropyrrolo[3′,2′:6,7]Cyclohepta[1,2-*d*][1,3]Thiazol-2-yl]Acetamide (**60**)

This compound was obtained by reaction of **47**. White solid; yield 73%, mp: 222.6–223.4 °C; IR: ν_max_ = 3408 (NH), 1713 (CO) cm^−1^; ^1^H NMR (200 MHz, CDCl_3_) δ 1.83 (s, 3H, CH_3_), 2.04–2.10 (m, 4H, 2 × CH_2_), 2.32–2.36 (m, 2H, CH_2_), 6.27 (s, 1H, H-7), 7.30–7.36 (m, 3H, H-3′, H-4′ and H-5′), 7.44 (s, 1H, H-8), 7.71 (d, *J* = 6.4 Hz, 2H, H-2′ and H-6′), 11.95 (s, 1H, NH); ^13^C NMR (50 MHz, CDCl_3_) δ 21.8 (q), 22.4 (t), 23.6 (t), 34.1 (t), 113.2 (d), 123.1 (d), 125.5 (s), 127.4 (2 × d), 128.7 (2 × d), 130.1 (s), 131.1 (s), 133.6 (d), 136.6 (s), 137.9 (s), 155.8 (s), 168.8 (s). Anal calcd for C_18_H_17_N_3_O_3_S_2_: C, 55.80; H, 4.42; N, 10.84. Found: C, 55.99; H, 4.27; N, 10.71.

##### Ethyl 2-(Acetylamino)-9-Methyl-4,5,6,9-Tetrahydropyrrolo[3′,2′:6,7]Cyclohepta[1,2-*d*][1,3]Thiazole-8-Carboxylate (**61**)

This compound was obtained by reaction of **49**. Pale yellow solid; yield: 62%, mp: 193.8–194.2 °C; IR: ν_max_ = 3399 (NH), 1701 (CO), 1597 (CO) cm^−1^; ^1^H NMR (200 MHz, CDCl_3_) δ 1.35 (t, *J* = 7.1 Hz, 3H, CH_3_), 1.88 (s, 3H, CH_3_), 2.06–2.14 (m, 2H, CH_2_), 2.61 (t, *J* = 6.3 Hz, 2H, CH_2_), 2.87 (t, *J* = 6.3 Hz, 2H, CH_2_), 4.12 (s, 3H, CH_3_), 4.28 (q, *J* = 7.1 Hz, 2H, CH_2_), 6.83 (s, 1H, H-7), 10.5 (s, 1H, NH); ^13^C NMR (50 MHz, CDCl_3_) δ 14.4 (q), 22.1 (q), 25.1 (t), 25.5 (t), 30.0 (t), 34.9 (q), 59.8 (t), 117.5 (d), 122.4 (s), 124.5 (s), 130.1 (s), 132.0 (s), 137.4 (s), 155.3 (s), 161.4 (s), 167.8 (s). Anal calcd for C_16_H_19_N_3_O_3_S: C, 57.64; H, 5.74; N, 12.60. Found: C, 57.48; H, 5.89; N, 12.77.

##### Ethyl 2-(Acetylamino)-9-(4-Methoxybenzyl)-4,5,6,9-Tetrahydropyrrolo[3′,2′:6,7]Cyclohepta[1,2-*d*][1,3]Thiazole-8-Carboxylate (**62**)

This compound was obtained by reaction of **50**. Yellow solid; yield 65 %, mp: 87.6–88.4 °C; IR: ν_max_ = 3403 (NH), 1696 (CO), 1557 (CO) cm^−1^; ^1^H NMR (200 MHz, CDCl_3_) δ 1.28 (t, *J* = 7.0 Hz, 3H, CH_3_), 1.92 (s, 3H, CH_3_), 2.11–2.19 (m, 2H, CH_2_), 2.61 (t, *J* = 6.3 Hz, 2H, CH_2_), 2.82 (t, *J* = 6.3 Hz, 2H, CH_2_), 3.69 (s, 3H, CH_3_), 4.20 (q, *J* = 7.0 Hz, 2H, CH_2_), 6.00 (s, 2H, CH_2_), 6.63 (d, *J* = 8.3 Hz, 2H, H-3′ and H-5′), 6.77 (d, *J* = 8.3 Hz, 2H H-2′ and H-6′), 6.91 (s, 1H, H-7), 10.11 (s, 1H, NH); ^13^C NMR (50 MHz, CDCl_3_) δ 14.3 (q), 22.5 (q), 24.7 (t), 25.2 (t), 30.8 (t), 48.5 (t), 55.1 (q), 59.8 (t), 113.5 (2 × d), 118.6 (d), 122.0 (s), 125.0 (s), 127.2 (2 × d), 130.2 (s), 131.7 (s), 132.0 (s), 137.6 (s), 155.2 (s), 158.2 (s), 161.0 (s), 167.8 (s). Anal calcd for C_23_H_25_N_3_O_4_S: C, 62.85; H, 5.73; N, 9.56. Found: C, 62.65; H, 5.58; N, 9.72.

##### Ethyl 2-(Acetylamino)-9-(3,5-Dimethoxybenzyl)-4,5,6,9-Tetrahydropyrrolo[3′,2′:6,7]Cyclohepta[1,2-*d*][1,3]Thiazole-8-Carboxylate (**63**)

This compound was obtained by reaction of **51**. Brown solid; Yield: 72%, mp: 70.2–71.0 °C; IR: ν_max_ = 3264 (NH), 1695 (CO), 1597 (CO) cm^−1^; ^1^H NMR (200 MHz, CDCl_3_) δ 1.27 (t, *J* = 7.1 Hz, 3H, CH_3_), 1.94 (s, 3H, CH_3_), 2.10–2.25 (m, 2H, CH_2_), 2.62 (t, *J* = 6.3 Hz, 2H, CH_2_), 2.83 (t, *J* = 6.3 Hz, 2H, CH_2_), 3.57 (s, 6H, 2 × CH_3_), 4.18 (q, *J* = 7.1 Hz, 2H, CH_2_), 5.98 (s, 2H, CH_2_), 6.04 (s, 2H, H-2′ and H-6′), 6.17 (s, 1H, H-4′), 6.93 (s, 1H, H-7), 9.96 (s, 1H, NH); ^13^C NMR (50 MHz, CDCl_3_) δ 14.3 (q), 22.6 (q), 24.8 (t), 25.3 (t), 30.7 (t), 49.1 (t), 55.0 (2 × q), 59.8 (t), 98.1 (d), 103.9 (2 × d), 118.8 (d), 122.2 (s), 124.9 (s), 130.1 (s), 132.0 (s), 137.8 (s), 142.4 (s), 154.9 (s), 160.6 (2 × s), 161.0 (s), 167.6 (s). Anal calcd for C_24_H_27_N_3_O_5_S: C, 61.39; H, 5.80; N, 8.95. Found: C, 61.57; H, 5.69; N, 9.11.

##### Ethyl 2-(Acetylamino)-9-(3,4,5-Trimethoxybenzyl)-4,5,6,9-Tetrahydropyrrolo[3′,2′:6,7]Cyclohepta[1,2-*d*][1,3]Thiazole-8-Carboxylate (**64**)

This compound was obtained by reaction of **52**. Pale brown solid; yield: 59%, mp: 73.1–73.8 °C; IR: ν_max_ = 3405 (NH), 1703 (CO), 1597 (CO) cm^−1^; ^1^H NMR (200 MHz, CDCl_3_) δ 1.30 (t, *J* = 7.1 Hz, 3H, CH_3_), 2.00 (s, 3H, CH_3_), 2.13–2.17 (m, 2H, CH_2_), 2.61–2.65 (m, 2H, CH_2_), 2.80–2.85 (m, 2H, CH_2_), 3.61 (s, 6H, 2 × CH_3_), 3.73 (s, 3H, CH_3_), 4.23 (q, *J* = 7.1 Hz, 2H, CH_2_), 6.08 (s, 2H, CH_2_), 6.13 (s, 2H, H-2′ and H-6′), 6.91 (s, 1H, H-7), 10.41 (s, 1H, NH); ^13^C NMR (50 MHz, CDCl_3_) δ 14.4 (q), 22.4 (q), 24.9 (t), 25.3 (t), 30.6 (t), 48.8 (t), 55.7 (2 × q), 59.9 (t), 60.7 (q), 103.2 (2 × d), 118.9 (d), 122.0 (s), 125.2 (s), 130.1 (s), 132.1 (s), 135.3 (s), 136.4 (s), 137.8 (s), 152.9 (2 × s), 155.3 (s), 161.2 (s), 168.0 (s). Anal calcd for C_25_H_29_N_3_O_6_S: C, 60.10; H, 5.85; N, 8.41. Found: C, 59.91; H, 5.98; N, 8.27.

##### Ethyl 2-(Acetylamino)-9-(2-Bromo-3,5-Dimethoxybenzyl)-4,5,6,9-Tetrahydropyrrolo[3′,2′:6,7]Cyclohepta[1,2-*d*][1,3]Thiazole-8-Carboxylate (**65**)

This compound was obtained by reaction of **53**. White solid; yield 69%, mp: 192.0–192.8 °C; IR: ν_max_ = 3413 (NH), 1704 (CO), 1593 (CO) cm^−1^; ^1^H NMR (200 MHz, CDCl_3_) δ 1.29 (t, *J* = 7.0 Hz, 3H, CH_3_), 1.94–2.12 (m, 5H, CH_3_ and CH_2_), 2.73–2.77 (m, 2H, CH_2_), 2.89–2.92 (m, 2H, CH_2_), 3.56 (s, 3H, CH_3_), 3.82 (s, 3H, CH_3_), 4.18 (q, *J* = 7.0 Hz, 2H, CH_2_), 5.44 (s, 1H, H-4′), 6.14 (s, 1H, CH_2_), 6.25 (s, 1H, H-6′), 6.95 (s, 1H, H-7), 9.16 (s, 1H, NH); ^13^C NMR (200 MHz, CDCl_3_) δ 14.3 (q), 23.1 (q), 26.2 (t), 26.7 (t), 28.2 (t), 51.0 (t), 55.1 (q), 56.2 (q), 59.8 (t), 97.1 (d), 101.1 (s), 103.4 (d), 118.9 (d), 122.3 (s), 124.8 (s), 129.8 (s), 131.8 (s), 137.2 (s), 142.1 (s), 153.5 (s), 156.1 (s), 159.8 (s), 160.9 (s), 167.5 (s). Anal calcd for C_24_H_26_BrN_3_O_5_S: C, 52.56; H, 4.78; N, 7.66. Found: C, 52.71; H, 4.59; N, 7.81.

##### General procedure for the synthesis of *N*-substituted[1,3]thiazole derivatives (**66**–**71**)

To a solution of the suitable 2-amine-thiazole **47**,**54**,**56** (0.46 mmol) in anhydrous 1,4-dioxane (3 mL), Et_3_N (0.07 mL, 0.51 mmol) and the suitable acyl chloride (0.69 mmol) were added and the reaction mixture was stirred at room temperature for 16 h. Then the reaction mixture was poured onto crushed ice. The precipitate was collected by filtration, dried and purified by column chromatography (dichloromethane).

##### 2-Methyl-*N*-[9-(Phenylsulfonyl)-4,5,6,9-Tetrahydropyrrolo[3′,2′:6,7]Cyclohepta[1,2-*d*][1,3]Thiazol-2-yl]Propanamide (**66**)

This compound was obtained by reaction of **47** with isobutyryl chloride. Light yellow solid; mp: 212–213 °C; yield 61%; IR: ν_max_ = 3250 (NH), 1686 (CO) cm^−1^; ^1^H NMR (200 MHz, CDCl_3_) δ 1.04 (s, 3H, CH_3_), 1.08 (s, 3H, CH_3_), 1.63–2.74 (m, 2H, CH_2_), 2.05–2.40 (m, 5H, 2 × CH_2_ and CH), 6.27 (d, *J* = 3.3 Hz, 1H, Ar), 7.31–7.52 (m, 4H, Ar), 7.70–7.74 (m, 2H, Ar), 10.89 (s, 1H, NH); ^13^C NMR (50 MHz, CDCl_3_) δ 19.2 (2 × q), 22.8 (t), 23.9 (t), 33.6 (t), 35.3 (d), 113.4 (d), 123.3 (d), 125.7 (s), 127.3 (2 × d), 128.7 (2 × d), 130.1 (s), 131.1 (s), 133.5 (d), 136.9 (s), 138.5 (s), 155.1 (s), 175.5 (s). Anal calcd for C_20_H_21_N_3_O_3_S_2_: C, 57.81; H, 5.09; N, 10.11. Found: C, 57.98; H, 4.91; N, 10.27.

##### *N*-(5-Chloro-2-Methoxyphenyl)-2-[(2-Methylpropanoyl)Amino]-4,5,6,9-Tetrahydropyrrolo[3′,2′:6,7]Cyclohepta[1,2-*d*][1,3]Thiazole-8-Carboxamide (**67**)

This compound was obtained by reaction of **54** with isobutyryl chloride. Yellow oil; yield 68%; IR: ν_max_ = 3422 (NH), 3250 (NH), 3193 (NH), 1686 (CO), 1646 (CO) cm^−1^; ^1^H NMR (200 MHz, CDCl_3_) δ 1.20 (s, 3H, CH_3_), 1.24 (s, 3H, CH_3_), 1.91–2.10 (m, 2H, CH_2_), 2.51–2.72 (m, 1H, CH), 2.77–2.92 (m, 4H, 2 × CH_2_), 3.84 (s, 3H, CH_3_), 6.44 (s, 1H, H-7), 6.72 (d, *J* = 8.7 Hz, 1H, Ar), 6.91 (dd, *J* = 8.7, 2.5 Hz, 1H, Ar), 8.15 (s, 1H, NH), 8.43 (d, *J* = 2.5 Hz, 1H, Ar), 9.29 (s, 1H, NH), 9.71 (s, 1H, NH); ^13^C NMR (50 MHz, CDCl_3_) δ 19.3 (2 × q), 23.9 (t), 27.4 (t), 28.4 (t), 35.5 (d), 56.1 (q), 110.7 (d), 112.0 (d), 119.4 (d), 122.4 (s), 122.6 (d), 123.3 (s), 125.5 (s), 126.2 (s), 128.7 (s), 129.4 (s), 137.1 (s), 146.3 (s), 154.6 (s), 158.6 (s), 174.7 (s). Anal calcd for C_22_H_23_ClN_4_O_3_S: C, 57.57; H, 5.05; N, 12.21. Found: C, 57.71; H, 4.92; N, 12.38.

##### *N*-(5-Chloro-2-Methoxyphenyl)-9-Methyl-2-[(2-Methylpropanoyl)Amino]-4,5,6,9-Tetrahydropyrrolo[3′,2′:6,7]Cyclohepta[1,2-*d*][1,3]Thiazole-8-Carboxamide (**68**)

This compound was obtained by reaction of **56** with isobutyryl chloride. Yellow oil; yield 80%; IR: ν_max_ = 3345 (NH), 3254 (NH), 1679 (CO) cm^−1^; ^1^H NMR (200 MHz, CDCl_3_) δ 1.22 (s, 3H, CH_3_), 1.25 (s, 3H, CH_3_), 2.07–2.19 (m, 2H, CH_2_), 2.68 (t, *J* = 6.5 Hz, 2H, CH_2_), 2.90 (t, *J* = 6.5 Hz, 2H, CH_2_), 3.92 (s, 3H, CH_3_), 4.07–4.25 (m, 4H, CH and CH_3_), 6.57 (s, 1H, H-7), 6.96–7.02 (m, 2H, Ar), 8.27 (s, 1H, Ar), 9.29 (s, 1H, NH), 13.37 (s, 1H, NH); ^13^C NMR (50 MHz, CDCl_3_) δ 19.2 (2 × q), 25.4 (t), 29.6 (t), 34.2 (t), 35.4 (d), 35.5 (q), 56.1 (q), 110.6 (d), 112.7 (d), 119.2 (d), 122.5 (d), 123.3 (s), 124.1 (s), 125.8 (s), 126.3 (d), 129.1 (s), 129.9 (s), 131.7 (s), 137.8 (s), 146.4 (s), 154.2 (s), 159.7 (s), 173.7 (s). Anal calcd for C_23_H_25_ClN_4_O_3_S: C, 58.40; H, 5.33; N, 11.85. Found: C, 58.57; H, 5.19; N, 11.99.

##### 2,2-Dimethyl-*N*-[9-(Phenylsulfonyl)-4,5,6,9-Tetrahydropyrrolo[3′,2′:6,7]Cyclohepta[1,2-*d*][1,3]Thiazol-2-yl]Propanamide (**69**)

This compound was obtained by reaction of **47** with trimethylacetyl chloride. White solid; mp: 67–68 °C; yield 62%; IR: ν_max_ = 3387 (NH), 1677 (CO) cm^−1^; ^1^H NMR (200 MHz, CDCl_3_) δ 1.32 (s, 9H, 3 × CH_3_), 1.99–2.12 (m, 2H, CH_2_), 2.37–2.46 (m, 4H, 2 × CH_2_), 6.22 (d, *J* = 3.3 Hz, 1H, Ar), 7.36–7.68 (m, 6H, Ar), 8.99 (s, 1H, NH); ^13^C NMR (50 MHz, CDCl_3_) δ 23.5 (t), 24.9 (t), 27.3 (3 × q), 31.9 (t), 39.1 (s), 114.4 (d), 123.9 (d), 126.5 (s), 126.7 (2 × d), 128.7 (2 × d), 129.7 (s), 131.2 (s), 133.2 (d), 137.4 (s), 139.8 (s), 153.5 (s), 176.1 (s). Anal calcd for C_21_H_23_N_3_O_3_S_2_: C, 58.72; H, 5.40; N, 9.78. Found: C, 58.90; H, 5.35; N, 9.89.

##### *N*-(5-Chloro-2-Methoxyphenyl)-2-[(2,2-Dimethylpropanoyl)Amino]-4,5,6,9-Tetrahydropyrrolo [3′,2′:6,7]Cyclohepta[1,2-*d*][1,3]Thiazole-8-Carboxamide (**70**)

This compound was obtained by reaction of **54** with trimethylacetyl chloride. Yellow solid; yield 63%; mp: 155–156 °C; IR: ν_max_ = 3423 (NH), 3395 (NH), 3255 (NH), 1666 (CO), 1655 (CO) cm^−1^; ^1^H NMR (200 MHz, DMSO-d_6_) δ 1.25 (s, 9H, 3 × CH_3_), 1.93–2.06 (m, 2H, CH_2_), 2.87 (t, *J* = 5.2 Hz, 2H, CH_2_), 3.01 (t, *J* = 5.2 Hz, 2H, CH_2_), 3.87 (s, 3H, CH_3_), 6.92 (s, 1H, H-7), 7.08–7.21 (m, 2H, Ar), 7.93 (d, *J* = 2.4 Hz, 1H, Ar), 9.20 (s, 1H, NH), 10.09 (s, 1H, NH), 11.65 (s, 1H, NH); ^13^C NMR (50 MHz, DMSO-d_6_) δ 23.8 (t), 26.5 (t), 26.6 (3 × q), 27.8 (t), 40.2 (s), 56.1 (q), 112.5 (d), 113.7 (d), 122.0 (s), 122.6 (d), 123.4 (s), 123.7 (s), 124.0 (d), 124.3 (s), 128.0 (s), 128.4 (s), 136.8 (s), 149.2 (s), 155.6 (s), 158.4 (s), 176.4 (s). Anal calcd for C_23_H_25_ClN_4_O_3_S: C, 58.40; H, 5.33; N, 11.85. Found: C, 58.61; H, 5.57; N, 11.69.

##### *N*-(5-Chloro-2-Methoxyphenyl)-2-[(2,2-Dimethylpropanoyl)Amino]-9-Methyl-4,5,6,9-Tetrahydropyrrolo[3′,2′:6,7]Cyclohepta[1,2-*d*][1,3]Thiazole-8-Carboxamide (**71**)

This compound was obtained by reaction of **56** with trimethylacetyl chloride. Yellow solid; yield 63%; mp: 280–281 °C; IR: ν_max_ = 3433 (NH), 3250 (NH), 1669 (CO) cm^−1^; ^1^H NMR (200 MHz, DMSO-d_6_) δ 1.26 (s, 9H, 3 × CH_3_), 1.95–2.09 (m, 2H, CH_2_), 2.61 (t, *J* = 6.4 Hz, 2H, CH_2_), 2.85 (t, *J* = 6.4 Hz, 2H, CH_2_), 3.87 (s, 3H, CH_3_), 4.17 (s, 3H, CH_3_), 6.87 (s, 1H, H-7), 7.07–7.20 (m, 2H, Ar), 8.03 (s, 1H, Ar), 8.88 (s, 1H, NH), 11.65 (s, 1H, NH); ^13^C NMR (50 MHz, DMSO-d_6_) δ 26.6 (3 × q), 27.1 (t), 29.0 (t), 29.2 (t), 48.9 (s), 56.2 (q), 64.6 (q), 112.4 (d), 118.1 (d), 121.3 (d), 123.8 (s), 124.9 (s), 126.8 (d), 131.6 (s), 133.7 (s), 137.4 (s), 143.7 (s), 148.6 (s), 154.7 (s), 159.5 (s), 165.9 (s), 179.3 (s). Anal calcd for C_24_H_27_ClN_4_O_3_S: C, 59.19; H, 5.59; N, 11.50. Found: C, 59.02; H, 5.73; N, 11.64.

### 3.2. Biology

The CF bronchial epithelial cells, CFBE41o-, with stable expression of F508del-CFTR [36] was stably transfected with the halide-sensitive yellow fluorescent protein, HS-YFP [37,38]. Cells were cultured with MEM plus 10% fetal calf serum, 2 mM L-glutamine, 100 U/mL penicillin, and 100 µg/mL streptomycin. For fluorescence-based measurement of CFTR activity, CFBE41o- cells were plated (50,000 cells/well) on clear-bottom 96-well black microplates (CLS3603, Corning). After 24 h after plating, cells were treated with test compounds (or vehicle, DMSO) at 37 °C for 24 h. At the time of assay, cells were washed three times with PBS (in mM: 137 NaCl, 2.7 KCl, 8.1 Na_2_HPO_4_, 1.5 KH_2_PO_4_, 1 CaCl_2_, and 0.5 MgCl_2_) to remove culture medium plus test compounds. Cells were then stimulated for 30 min with forskolin (20 µM) plus genistein (50 µM) in 60 µL PBS. The microplate was subsequently transferred to a microplate reader (FluoStar Galaxy; BMG Labtech) equipped with high-quality excitation (ET500/20X: 500 ± 10 nm) and emission (ET535/30M: 535 ± 15 nm) filters for YFP (Chroma Technology). Assay consisted of a continuous 14-s fluorescence reading in each well, with injection at 2 s of 165 µL of an iodide-rich solution (modified PBS, with Cl^−^ replaced by I^−^; final I^−^ concentration in the well: 100 mM). Fluorescence was read every 0.2 s, with 20 excitation flashes per time point. Data were normalized to the initial background-subtracted fluorescence. To determine fluorescence quenching rate (QR) reflecting the extent of I^−^ influx, the data points corresponding to the final 11 s of the fluorescence reading for each well were fitted with an exponential function (Igor software, Wavemetrics) to extrapolate initial slope (dF/dt).

## 4. Conclusions

Thirty-five compounds having a pyrrolothiazole structure were synthesized to assess their ability in the rescue of chloride channel function of F508del −CFTR. Our study led to the identification of one compound with promising activity as a corrector. This compound having a six atom carbocyle ring and bearing a pivalamide group at the thiazole moiety and a 5-chloro-2-methoxyphenyl carboxamide at the pyrrole ring had improved activity compared to other substitutions. Any manipulation of peripheral groups in the pyrrolothiazole structure led to a decrease of the activity, as in the case of the isopropyl amide analogue or of the cyclohepta analogue, or to a loss of activity.

Compared to the parent constrained bithiazoles, replacement of the thiazole ring with the pyrrole one did not lead to an improvement in activity. However, the presence of 5-chloro-2-methoxyphenyl and the pivalamide groups is confirmed as an important structural requirement to obtain activity.

The drug discovery is a long process, requiring subsequent advancements of knowledge and synthetic strategies to select lead scaffolds, to reach good activity and selectivity of final compounds. Although, in this study we found corrector activity only for one compound, the pyrrolothiazole **44**, our results may represent the starting point for the development of a new series of CFTR correctors. This preliminary result gives in fact important insight to further investigate this class of compounds in the near future, paving the way to new tricyclic structures containing the pyrrole moiety as valuable correctors.

Intriguingly, compound **44** showed additive effects when combined with VX-809 but not with VX-661. This is a surprising result since these two correctors are structurally similar and are believed to act with a similar mechanism of action.

Our work offers the basis for further structural modifications to explore the SAR and the chemical space around the tricyclic core. Other positional isomers could be investigated, maintaining the decoration at the pyrrole and thiazole rings. Overall, these results provide us a basis for selection and optimization of lead candidates and it could represent a resource for medicinal chemists who are interested in this field, to explore diverse pharmacophore structural space for enhancing the discovery of new compounds.

## Figures and Tables

**Table 1:** Overview of *α*-bromo-ketones **21**–**36**.

Compound	Method	R	R^1^	*n*	Compound	Method	R	R^1^	*n*
**21**	A	SO_2_Ph	H	1	**29**	A	3,4,5-(MeO)_3_Bn	COOEt	2
**22**	-	H	H	2	**30**	A	2-Br-3,5-(MeO)_2_Bn	COOEt	2
**23**	A	SO_2_Ph	H	2	**31**	B	H	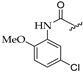	1
**24**	B	COPh	H	2	**32**	B	Me	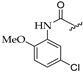	1
**25**	A	H	COOEt	2	**33**	B	H	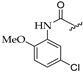	2
**26**	A	Me	COOEt	2	**34**	B	H	CONHtBu	2
**27**	A	4-MeOBn	COOEt	2	**35**	B	Me	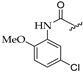	2
**28**	A	3,5-(MeO)_2_Bn	COOEt	2	**36**	B	Me	CONHtBu	2

**Table 2:**
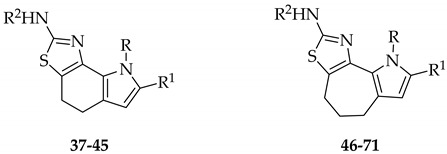
[1,3]Thiazolo[5,4-g]indole-2-amines 37–45 and pyrrolo[3′,2′:6,7]cyclohepta[1,2-d][1,3]thiazol-2-amines 46–71.

Cmp	R	R^1^	R^2^	Yield	Cmp	R	R^1^	R^2^	Yield
**37**	SO_2_Ph	H	H	90%	**55**	H	*t*BuNHCO	H	74%
**38**	H	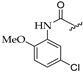	H	70%	**56**	Me	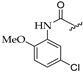	H	93%
**39**	Me	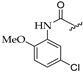	H	93%	**57**	Me	*t*BuNHCO	H	90%
**40**	SO_2_Ph	H	*i*PrCO	60%	**58**	H	*t*BuNHCO	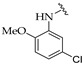	65%
**41**	H	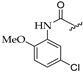	*i*PrCO	68%	**59**	Me	*t*BuNHCO	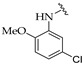	85%
**42**	Me	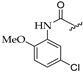	*i*PrCO	81%	**60**	SO_2_Ph	H	Acetyl	73%
**43**	SO_2_Ph	H	*t*BuCO	65%	**61**	Me	COOEt	Acetyl	62%
**44**	H	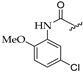	*t*BuCO	65%	**62**	4- OMe-Bn	COOEt	Acetyl	65%
**45**	Me	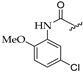	*t*BuCO	78%	**63**	3,5- (OMe)_2_-Bn	COOEt	Acetyl	72%
**46**	H	H	H	64%	**64**	3,4,5- (OMe)_3_-Bn	COOEt	Acetyl	59%
**47**	SO_2_Ph	H	H	64%	**65**	2-Br-3,5- (OMe)_2_-Bn	COOEt	Acetyl	69%
**48**	H	COOEt	H	62%	**66**	SO_2_Ph	H	*i*PrCO	61%
**49**	Me	COOEt	H	60%	**67**	H	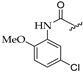	*i*PrCO	68%
**50**	4- OMe-Bn	COOEt	H	69%	**68**	Me	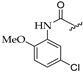	*i*PrCO	80%
**51**	3,5- (OMe)_2_-Bn	COOEt	H	58%	**69**	SO_2_Ph	H	*t*BuCO	62%
**52**	3,4,5- (OMe)_3_-Bn	COOEt	H	92%	**70**	H	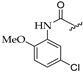	*t*BuCO	63%
**53**	2-Br-3,5- (OMe)_2_-Bn	COOEt	H	53%	**71**	Me	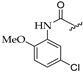	*t*BuCO	63%
**54**	H	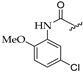	H	96%					

## Sample Availability

Samples of the compounds are available from the authors upon reasonable request.
